# Peripherally administered androgen receptor–targeted antisense oligonucleotide rescues spinal pathology in a murine SBMA model

**DOI:** 10.1172/JCI182955

**Published:** 2025-08-28

**Authors:** Changwoo Lee, Zhigang Yu, Curtis J. Kuo, Leon Tejwani, Rosalie M. Grijalva, Eunwoo Bae, Hien T. Zhao, Janghoo Lim, Andrew P. Lieberman

**Affiliations:** 1 Interdepartmental Neuroscience Program, Department of Neuroscience, Yale School of Medicine, New Haven, Connecticut, USA.; 2 Department of Pathology and; 3 Cellular and Molecular Biology Graduate Program, Medical Scientist Training Program, University of Michigan Medical School, Ann Arbor, Michigan, USA.; 4 Department of Genetics, Yale School of Medicine, New Haven, Connecticut, USA.; 5 Ionis Pharmaceuticals Inc., Carlsbad, California, USA.; 6 Department of Neuroscience, Yale School of Medicine, New Haven, Connecticut, USA.

**Keywords:** Neuroscience, Therapeutics, Neurodegeneration, Neuromuscular disease

## Abstract

Degeneration of the neuromuscular system is a characteristic feature of spinal and bulbar muscular atrophy (SBMA), a CAG/polyglutamine (polyQ) expansion disorder caused by mutation in the androgen receptor (AR). Using a gene-targeted mouse model of SBMA, AR113Q mice, we demonstrate age-dependent degeneration of the neuromuscular system that initially manifests with muscle weakness and atrophy and progresses to include denervation of neuromuscular junctions and lower motor neuron soma atrophy. Using this model, we tested the hypothesis that therapeutic intervention targeting skeletal muscle during this period of disease progression arrests degeneration of the neuromuscular system. To accomplish this, *AR*-targeted antisense oligonucleotides were administered subcutaneously to symptomatic AR113Q mice to reduce expression of polyQ AR in peripheral tissues but not in the spinal cord. This intervention rescued muscle atrophy, neuromuscular junction innervation, lower motor neuron soma size, and survival in aged AR113Q mice. Single-nucleus RNA sequencing revealed age-dependent transcriptional changes in the AR113Q spinal cord during disease progression, which were mitigated by peripheral *AR* gene silencing. Our findings underscore the intricate interplay between peripheral tissues and the central nervous system in SBMA and emphasize the therapeutic effectiveness of peripheral gene knockdown in symptomatic disease.

## Abstract 2

Peripheral administration of AR-targeted ASOs rescue disease in SBMA mice and show unanticipated benefits on the spinal cord transcriptional landscape, providing mechanistic insights into pathogenesis.

## Introduction

Therapeutic approaches for age-dependent protein aggregation neurodegenerative disorders remain elusive, in part because of an incomplete understanding of disease mechanisms. Inherited degenerative disorders such as those caused by CAG/polyglutamine (polyQ) tract expansions present well-defined genetic targets (1), but even in this context, the identification of disease-modifying therapies has proven challenging. This is exemplified by spinal and bulbar muscular atrophy (SBMA), a degenerative disorder of the neuromuscular system caused by a polyQ tract expansion in the androgen receptor (AR). SBMA is characterized by skeletal muscle weakness and atrophy that are accompanied by the progressive loss of lower motor neurons in the spinal cord and brain stem (2). This degeneration of the neuromuscular system along with signs of partial androgen insensitivity is a disease hallmark and is often accompanied by additional phenotypes including sensory nerve involvement (3, 4). Notably, ligand-dependent nuclear translocation of the polyQ AR is required for pathogenesis (5, 6), underlying the restriction of substantial disease manifestations to males but not females who inherit a mutant *AR* allele.

Mouse models used to study the SBMA pathogenic cascade have implicated skeletal muscle as an early site of disease manifestations and a potential therapeutic target (7, 8). Indeed, mice in which much of human *AR* exon 1 containing a long CAG repeat was targeted to mouse *Ar* exon 1 (AR113Q mice) (9) exhibit skeletal muscle atrophy that precedes spinal cord pathology (10). While this muscle atrophy is driven in part by functional impairment of the transcription factor MEF2 mediated by the polyQ AR (11), less is known about mechanisms leading to lower motor neuron toxicity. Nonetheless, important contributions of skeletal muscle to spinal cord disease have been suggested previously (12–14). Peripheral administration of *AR*-targeted antisense oligonucleotides (ASOs) to presymptomatic and early-symptomatic AR113Q mice and BAC transgenic SBMA mice rescues disease manifestations by targeting peripheral tissues without altering polyQ AR expression in spinal cord (13, 15). These findings are complemented by studies demonstrating that conditional deletion of the polyQ AR only in skeletal muscle of BAC transgenic SBMA mice also rescues disease phenotypes (12).

These observations, and concurrent studies in individuals with SBMA that identified early alterations in skeletal muscle biomarkers (16, 17), underlie a current model of disease pathogenesis in which degeneration of the neuromuscular system manifests initially in skeletal muscle and progresses to involve the spinal cord (7). An important hypothesis emerging from this model is that, in symptomatic disease, there exists a window of opportunity for therapeutic intervention during which targeting skeletal muscle will provide benefit against the degenerative process that affects the entire neuromuscular system. Here, we sought to test this notion. We demonstrate that symptomatic AR113Q male mice at 6 months exhibit significant skeletal muscle atrophy and impaired motor function, but not neuromuscular junction (NMJ) denervation or spinal lower motor neuron pathology. By 12 months, both NMJ and motor neuron pathology is present along with atrophic muscles. Subcutaneous administration of *AR*-targeted ASO to AR113Q mice during this 6-month window rescues these pathologies and resets gene expression changes in spinal cord, as detected by single-nucleus RNA sequencing (snRNA-Seq). Our studies demonstrate benefits of peripheral ASO administration in aged symptomatic mice, present an unbiased characterization of transcriptional changes in the SBMA spinal cord at single-cell resolution, and demonstrate unanticipated benefits of peripheral ASO administration for the spinal cord transcriptional landscape, thereby providing mechanistic insights into disease pathogenesis.

## Results

### Symptomatic AR113Q mice at 26 weeks exhibited muscle atrophy but not motor neuron pathology.

**Figure 1:**
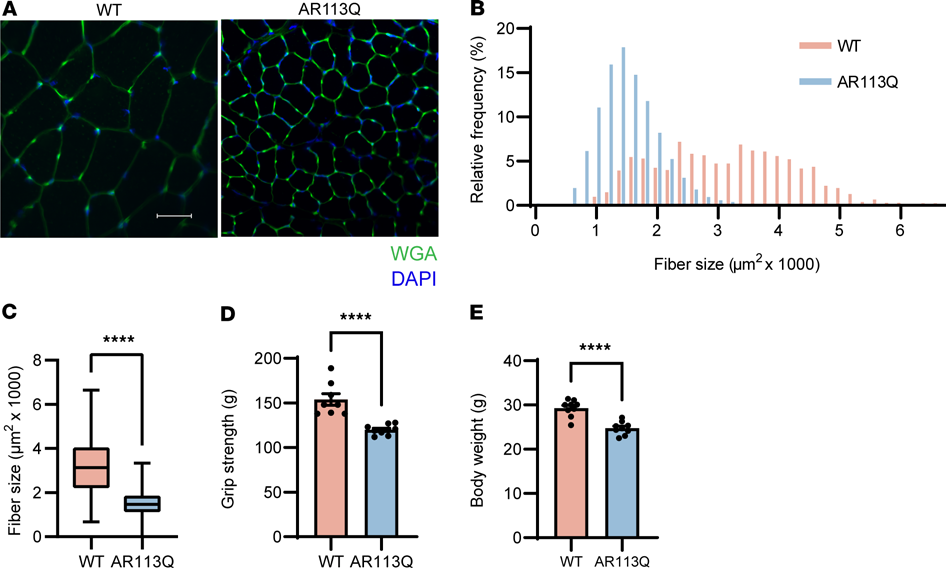
AR113Q males exhibit skeletal muscle atrophy and weakness at 26 weeks. (**A**) TA muscle fibers from WT or AR113Q males were visualized by FITC–wheat germ agglutinin (WGA). Nuclei were stained with DAPI (blue). Scale bar: 50 μm. (**B** and **C**) Fiber size quantified as a histogram of frequency distribution (**B**) and box plot (**C**). *n* = 3 mice per genotype, >100 fibers per mouse. In **C**, the box is the interquartile range, the center line is the median, and the whiskers are the minimum and maximum values. ****P < 0.0001 by unpaired Student’s *t* test, *F* = 5.314, degrees of freedom (df) = 1,627. (**D**) Grip strength of WT and AR113Q mice (WT, *n* = 8; AR113Q, *n* = 9). Data are mean ± SEM. ****P < 0.0001 by unpaired Student’s *t* test, *F* = 11.07, df = 7. (**E**) Body weight of WT and AR113Q mice (WT, *n* = 9; AR113Q, *n* = 9). Data are mean ± SEM. ****P < 0.0001 by unpaired Student’s *t* test, *F* = 1.642, df = 8.

We conducted a longitudinal study in aged, symptomatic AR113Q male mice to assess the effects of knocking down polyQ AR expression in peripheral tissues. AR113Q male mice at 26 weeks displayed significant atrophy of the tibialis anterior (TA), a disease-relevant hind-limb muscle (Figure 1, A–C). These mice also exhibited significantly diminished neuromuscular function as assessed by grip strength (Figure 1D) as well as decreased body weight (Figure 1E).

**Figure 2:**
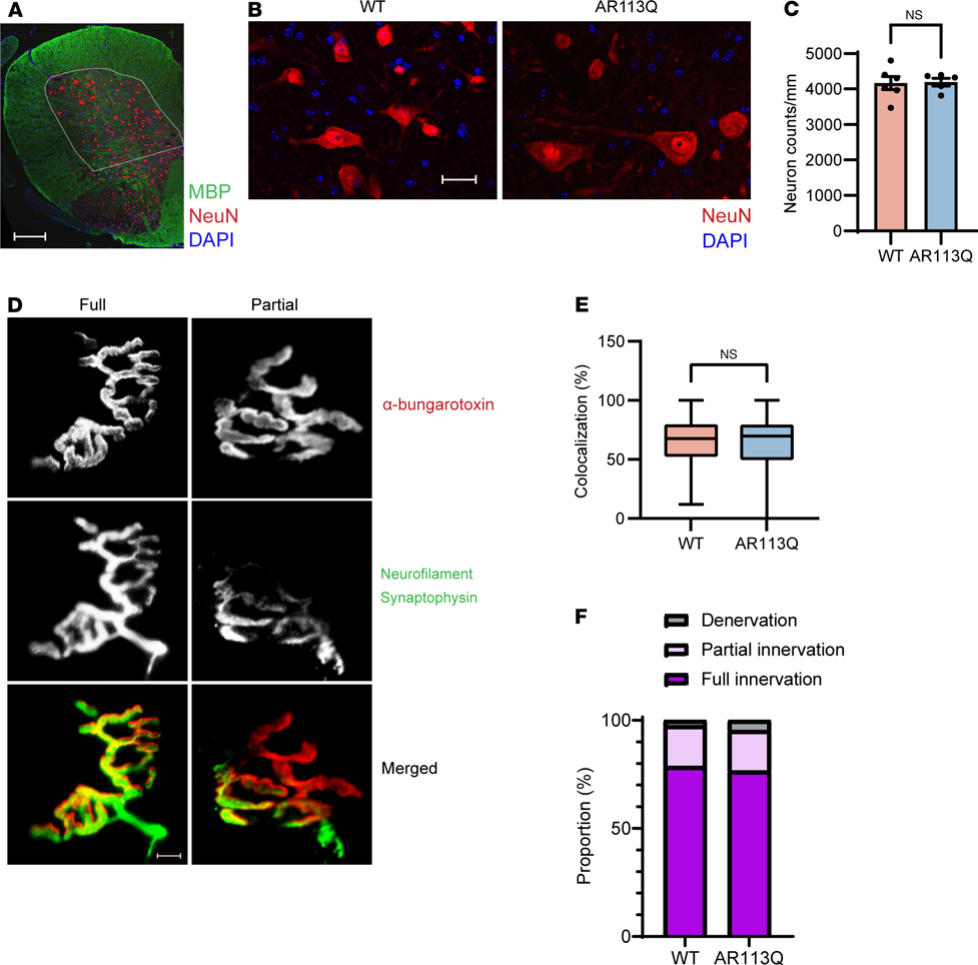
AR113Q males exhibit no significant spinal cord or NMJ pathology at 26 weeks. (**A**) Immunohistochemistry of spinal cord lumbar enlargement. NeuN is shown in red, MBP in green. White line designates area of neuron quantification. Scale bar: 250 μm. (**B**) Large neurons in anterior spinal cord lumbar enlargement. NeuN is shown in red, DAPI in blue. Scale bar: 25 μm. (**C**) Density of large neurons in anterior spinal cord lumbar enlargement (WT, *n* = 6; AR113Q, *n* = 5). Data are mean ± SEM. ns, not significant by unpaired Student’s *t* test; *F* = 3.372, df = 5. (**D**) NMJs in TA muscle were visualized by immunofluorescence staining for α-bungarotoxin (red) and neurofilament plus synaptophysin (green). Representative images of NMJs exhibiting full innervation (colocalization ≥50%) and partial innervation (colocalization >20% to <50%). Scale bar: 10 μm. (**E** and **F**) NMJ innervation quantified as a box plot (**E**) or stacked bar graph (**F**). *n* = 3 mice per genotype, 100 NMJs per mouse. In **E**, the box is the interquartile range, the center line is the median, and the whiskers are the minimum and maximum values. NS, not significant by unpaired Student’s *t* test; *F* = 1.394, df = 299.

Unbiased stereology was used to quantify the density of large neurons (soma diameter >30 μm) in the ventral gray matter of the lumbar enlargement as visualized in serial transverse sections stained for NeuN (Figure 2, A–C). No significant difference in neuron number was detected between wild-type (WT) and AR113Q males. Similarly, staining for choline acetyltransferase (ChAT) highlighted large lower motor neurons in spinal cord of WT and AR113Q males (Supplemental Figure 1A; supplemental material available online with this article; https://doi.org/10.1172/JCI182955DS1). Additionally, NMJs of the TA were stained for presynaptic (neurofilament, synaptophysin) and postsynaptic markers (α-bungarotoxin) (Figure 2D), and colocalization of staining was assessed to determine the extent of innervation. No significant differences were observed between WT and AR113Q muscle (Figure 2, E and F), as more than 75% of NMJs were fully innervated in both genotypes and only a small fraction (<5%) were denervated. Our analyses indicated that 26-week AR113Q males exhibited significant muscle weakness and atrophy but did not display spinal cord or NMJ pathology. We enrolled these symptomatic animals in an intervention trial targeting polyQ AR expression in peripheral tissues to assess effects on SBMA disease progression.

### A modified AR-targeted ASO for subcutaneous administration.

We sought to selectively knock down polyQ AR expression in peripheral tissues. This approach extends prior work demonstrating that subcutaneously administered *AR*-targeted ASO delivered to AR113Q males from 8 to 26 weeks of age ameliorates disease phenotypes (13, 15). These prior studies used a 16-mer 3-10-3 constrained ethyl (cEt) gapmer ASO. We initially tested alternative *AR*-targeted ASO formulations containing the identical nucleotide sequence that was used previously, but with a modified chemical backbone (4-8-4 mixed cEt/methoxyethyl gapmer) that is particularly well tolerated in mice (Supplemental Figure 2, ASO1) (18). We additionally tested an ASO with this same sequence and chemistry, but conjugated to palmitic acid via a phosphodiester linkage at the 5′ end. This fatty acid conjugate enhances association of the ASO with plasma proteins, with the goal of facilitating transcytosis across the capillary endothelium into tissues such as skeletal muscle (Supplemental Figure 2, ASO2) (18, 19). Palmitic acid–conjugated ASOs have been demonstrated to be safe, efficacious, and well tolerated (19–22). ASOs were administered weekly to WT males by subcutaneous administration starting at 8 weeks of age and continuing for 4 weeks. Analysis of the TA, soleus, and diaphragm demonstrated dose-dependent knockdown of *Ar* mRNA following treatment with either ASO (Supplemental Figure 2). The palmitic acid–conjugated formulation at a dose of 25 mg/kg triggered significant target knockdown of more than 60% in all 3 muscles; this effect was equivalent to target knockdown achieved by administration of the non-conjugated formulation at 50 mg/kg (Supplemental Figure 2). Dosing with higher concentrations of the palmitic acid–conjugated ASO yielded marginally more *Ar* mRNA knockdown in TA but not liver (Supplemental Figure 2). Therefore, we elected to treat AR113Q males with the palmitic acid–conjugated formulation at a dose of 25 mg/kg, administered subcutaneously once per week.

To assess the safety, tolerability, and target engagement of this ASO formulation, WT males received subcutaneous administration of palmitic acid–conjugated, *AR*-targeted or non-targeted ASO at 25 mg/kg/wk from 26 until 52 weeks (Supplemental Figure 3). No significant change in body weight was observed, although a trend toward decreased body weight was noted. Administration of *AR*-targeted ASO yielded a small decrease in grip strength at 52 weeks, accompanied by diminished TA weight and muscle fiber size as likely consequences of partial loss of AR function. *AR*-targeted ASO resulted in significantly diminished AR protein levels in TA and liver, without altering AR protein expression in spinal cord (Supplemental Figure 4). Moreover, bulk RNA-Seq of lumbar spinal cord from mice receiving *AR*-targeted versus non-targeted ASO at 52 weeks revealed only 6 differentially expressed genes (Supplemental Figure 5), indicating that peripheral *Ar* gene knockdown in WT mice had minimal effects on spinal cord gene expression. Assessment of serum markers of liver and kidney function in 52-week mice showed no values outside the normal range, except for marginally elevated levels of blood urea nitrogen (Supplemental Figure 6). We conclude that this ASO formulation has favorable characteristics for long-term testing in AR113Q mice.

### AR-targeted ASO rescued muscle atrophy and survival in symptomatic AR113Q mice.

**Figure 3:**
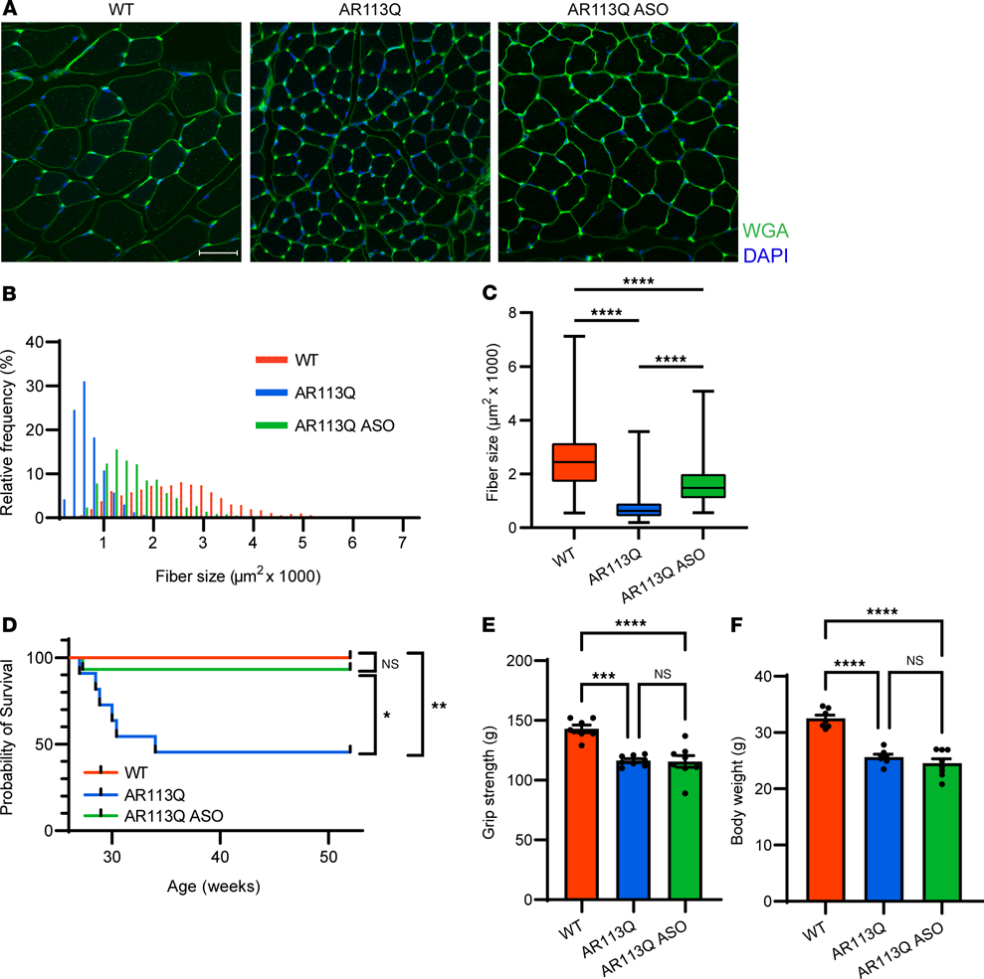
Peripheral ASO administration from 26 to 52 weeks rescues survival and ameliorates skeletal muscle atrophy. AR113Q males at 26 weeks were given ASO (25 mg/kg body weight) or vehicle subcutaneously, once per week until 52 weeks. (**A**) TA muscle fibers from WT, AR113Q, or AR113Q plus ASO males were visualized by FITC-WGA. Nuclei were stained with DAPI (blue). Scale bar: 50 μm. (**B** and **C**) TA fiber size quantified as a histogram of frequency distribution (**B**) and box plot (**C**). In **C**, the box is the interquartile range, the center line is the median, and the whiskers are the minimum and maximum values. *n* = 3 mice per group, >100 fibers per mouse. ****P < 0.0001 by 1-way ANOVA with Tukey’s multiple-comparison test, *F* = 424.7, df = 2. (**D**) Survival curve of WT, AR113Q, and AR113Q plus ASO mice (WT, *n* = 15; AR113Q, *n* = 11; AR113Q + ASO, *n* = 15). NS, not significant; *P < 0.05, **P < 0.01 by log-rank test with Bonferroni’s correction, χ^2^ = 16.07, df = 2. (**E**) Grip strength of WT, AR113Q, and AR113Q plus ASO mice at 52 weeks (WT, *n* = 7; AR113Q, *n* = 8; AR113Q + ASO, *n* = 8). Data are mean ± SEM. ***P < 0.001, ****P < 0.0001 by 1-way ANOVA with Tukey’s multiple-comparison test, *F* = 18.72, df = 2. (**F**) Body weight of WT, AR113Q, and AR113Q plus ASO mice at 52 weeks (WT, *n* = 7; AR113Q, *n* = 8; AR113Q + ASO, *n* = 8). Data are mean ± SEM. ****P < 0.0001 by 1-way ANOVA with Tukey’s multiple-comparison test, *F* = 39.40, df = 2.

Symptomatic AR113Q males were randomized to treatment versus vehicle control groups. The treatment group received weekly subcutaneous administration of *AR*-targeted ASO from 26 until 52 weeks, a time interval during which AR113Q mice exhibited a significant, age-dependent exacerbation of neuromuscular degeneration (Supplemental Figure 7). Therapeutic intervention resulted in significant, albeit partial, rescue of TA muscle atrophy in comparison with vehicle-treated 52-week AR113Q males (Figure 3, A–C). Moreover, ASO administration rescued AR113Q survival to levels equivalent to those of WT mice, while approximately 50% of control AR113Q males died by the study’s endpoint (Figure 3D). Notably, peripheral ASO administration did not rescue either grip strength or body weight of AR113Q males (Figure 3, E and F). These findings indicate that peripheral administration of *AR*-targeted ASO after symptom onset is sufficient to partially rescue SBMA mice.

### Peripheral ASO rescued spinal cord and NMJ pathology.

**Figure 4:**
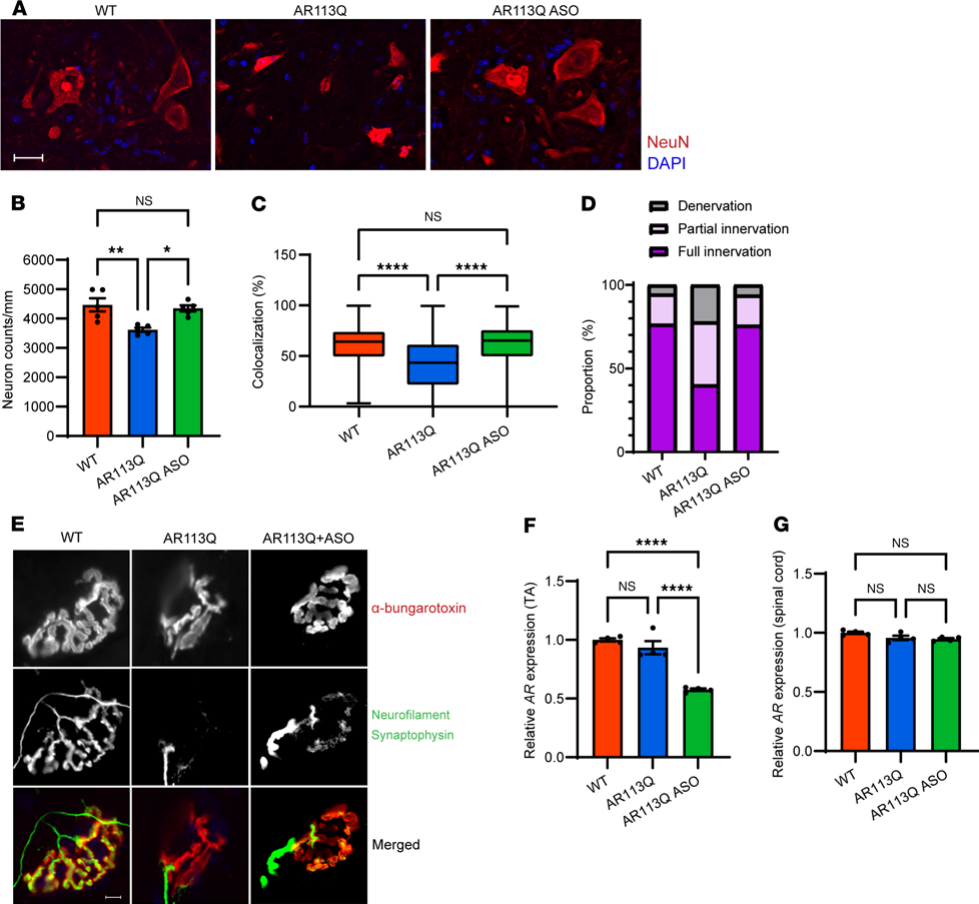
Spinal cord and NMJ pathology of 52-week AR113Q mice is rescued by peripheral ASO administration. (**A**) Large neurons in anterior spinal cord lumbar enlargement. NeuN is shown in red, DAPI in blue. Scale bar: 25 μm. (**B**) Density of large neurons in anterior spinal cord lumbar enlargement from WT (*n* = 5), AR113Q (*n* = 5), and AR113Q plus ASO (*n* = 5) mice. Data are mean ± SEM. ns, not significant; *P < 0.05, **P < 0.01 by 1-way ANOVA with Tukey’s multiple-comparison test, *F* = 9.38, df = 2. (**C**–**E**) NMJs in TA muscle were visualized by immunofluorescence staining for α-bungarotoxin and neurofilament plus synaptophysin. Innervation quantified as a box plot (**C**) or stacked bar graph (**D**). In **C**, the box is the interquartile range, the center line is the median, and the whiskers are the minimum and maximum values. One hundred NMJs per mouse. ns, not significant; ****P < 0.0001 by 1-way ANOVA with Tukey’s multiple-comparison test, *F* = 82.00, df = 2. (**E**) Representative images of NMJs exhibiting full innervation (colocalization ≥50%) from WT muscle, denervation (colocalization ≤ 20%) from AR113Q muscle, and partial innervation (colocalization >20% to <50%) from AR113Q plus ASO muscle. Scale bar: 10 μm. (**F** and **G**) Relative *Ar* mRNA by quantitative PCR in TA muscle (**F**) and spinal cord (**G**) of WT, AR113Q, and AR113Q plus ASO mice (*n* = 4 per group). Data are mean ± SEM. NS, not significant; ****P < 0.0001 by 1-way ANOVA with Tukey’s multiple-comparison test. In **E**, *F* = 45.43, df = 2; in **F**, *F* = 4.347, df =2.

We evaluated the spinal cord and NMJ of 52-week AR113Q mice to investigate potential non-cell-autonomous effects of peripheral ASO administration on central nervous system phenotypes. Quantification of large neurons (soma diameter >30 μm) revealed a significant, age-dependent reduction in neuron density in AR113Q spinal cord from 26 to 52 weeks (Supplemental Figure 7B). At 52 weeks, AR113Q males showed a significant reduction of large-neuron density compared with WT males, reflecting either soma atrophy or neuron loss. Remarkably, this pathology was completely rescued by peripheral ASO administration (Figure 4, A and B). Staining for ChAT highlighted atrophic neuronal soma in AR113Q spinal cord and large lower motor neurons in AR113Q cord following peripheral ASO administration (Supplemental Figure 1B). Furthermore, AR113Q males showed a substantial increase in the fraction of partially innervated and denervated NMJs from 26 to 52 weeks (Supplemental Figure 7, C and D). At 52 weeks, AR113Q males displayed a marked increase in the fraction of partially innervated and denervated NMJs compared with WT. This was accompanied by a decrease in the fraction of fully innervated NMJs from approximately 75% in WT males to approximately 40% in AR113Q males. These pathological changes were fully rescued in ASO-treated AR113Q males (Figure 4, C–E). Importantly, this rescue of spinal cord and NMJ pathology occurred concurrently with a significant knockdown of *Ar* mRNA in skeletal muscle (Figure 4F) but not spinal cord (Figure 4G) following peripheral ASO administration, suggesting that beneficial therapeutic effects of targeting of skeletal muscle extended to impact cells within the spinal cord.

### Single-nucleus RNA sequencing of AR113Q mouse spinal cord.

**Figure 5:**
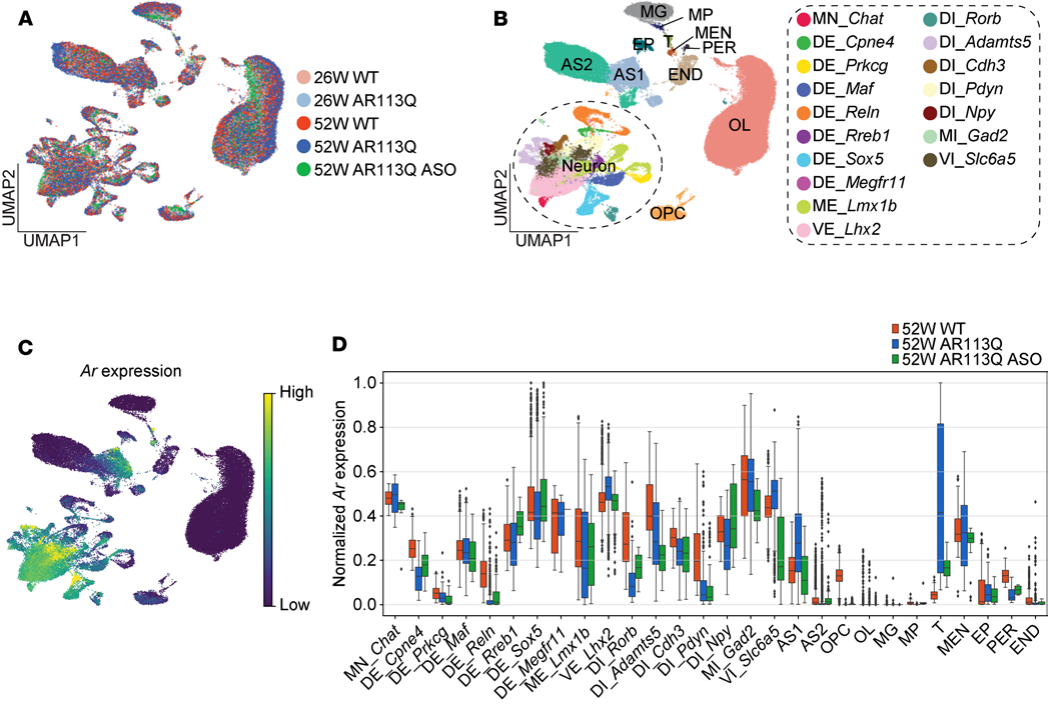
Single-nucleus RNA sequencing of AR113Q lumbar cord. (**A** and **B**) UMAP embeddings of 75,870 nuclei by time point, genotype, and ASO treatment (**A**) and by cell type (**B**). Identified cell types include *Chat^+^* motor neurons (MN_*Chat*), 7 families of dorsal excitatory neurons (DE_*Cpne4*, DE_*Prkcg4*, DE_*Maf*, DE_*Reln*, DE_*Rreb1*, DE_*Sox5*, and DE_*Megfr11*), medial excitatory neurons (ME_*Lmx1b*), ventral excitatory neurons (VE_*Lhx2*), 5 families of dorsal inhibitory neurons (DI_*Rorb*, DI_*Adamts5*, DI_*Cdh3*, DI_*Pdyn*, and DI_*Npy*), medial inhibitory neurons (MI_*Gad2*), ventral inhibitory neurons (VI_*Slc6a5*), astrocytes 1 (AS1), astrocytes 2 (AS2), oligodendrocyte progenitor cells (OPC), oligodendrocytes (OL), microglia (MG), macrophages (MP), T cells (T), meningothelial cells (MEN), ependymal cells (EP), pericytes (PER), and endothelial cells (END). (**C**) UMAP embedding showing the relative expression of *Ar*. (**D**) Box plots showing normalized expression of *Ar* in each cell type in the 52-week groups. Boxes indicate the interquartile range, center lines are the median, and the whiskers and individual points were drawn by the Tukey’s method.

To directly assess gene expression changes in AR113Q spinal cord and the effects of peripheral ASO administration, we performed snRNA-Seq. Recent spatial transcriptomic analyses identified a diverse array of cell types in the mouse spinal cord (23). Notably, the transcriptional impact of the polyQ AR on these individual cell types has not been previously explored. To investigate the longitudinal transcriptional profile of the SBMA lumbar cord and the effects of peripheral ASO administration at single-cell resolution, we performed snRNA-Seq of the lumbar spinal enlargement isolated from 26-week and 52-week AR113Q mice and their WT littermates along with 52-week ASO-treated AR113Q mice (Supplemental Table 1). This enabled assessment of transcriptional changes in symptomatic mice at discrete time points prior to the occurrence of NMJ or lower motor neuron pathology, after their occurrence, and in the setting of therapeutic ASO administration. Integration of data across individual samples followed by thorough pre-processing and quality control yielded a total of 75,870 high-quality nuclei (Figure 5A, Supplemental Figure 8A, and Supplemental Table 1). Major neuronal, glial, and other cell types in the spinal cord were assigned to nucleus clusters of similar transcriptional identities based on expression of established marker genes (Figure 5B and Supplemental Figure 8, B and G) (23). The neuronal types included *Chat^+^* motor neurons (MN_*Chat*), 7 families of dorsal excitatory neurons expressing *Cpne4*, *Prkcg4*, *Maf*, *Reln*, *Rreb1*, *Sox5*, or *Megfr11* (DE_*Cpne4*, DE_*Prkcg4*, DE_*Maf*, DE_*Reln*, DE_*Rreb1*, DE_*Sox5*, and DE_*Megfr11*), *Lmx1b^+^* medial excitatory neurons (ME_*Lmx1b*), *Lhx2^+^* ventral excitatory neurons (VE_*Lhx2*), 5 families of dorsal inhibitory neurons expressing *Rorb*, *Adamts5*, *Cdh3*, *Pdyn*, or *Npy* (DI_*Rorb*, DI_*Adamts5*, DI_*Cdh3*, DI_*Pdyn*, and DI_*Npy*), *Gad2^+^* medial inhibitory neurons (MI_*Gad2*), and *Slc6a5^+^* ventral inhibitory neurons (VI_*Slc6a5*). Glial cells included activated/fibrous *Gfap^hi^*, *Aqp4^hi^*, *Slc7a10^lo^* astrocytes (AS1), regular/protoplasmic *Gfap^lo^*, *Aqp4^lo^*, *Slc7a10^hi^* astrocytes (AS2), oligodendrocyte progenitor cells (OPC), oligodendrocytes (OL), and microglia (MG). Other cell types included macrophages (MP), T cells (T), meningothelial cells (MEN), ependymal cells (EP), pericytes (PER), and endothelial cells (END).

We compared motor neuron abundance among genotypes and treatment groups within each time point because of the observed reduction of large-neuron density in AR113Q mice and rescue by peripheral ASO administration at 52 weeks (Figure 4, A and B). Interestingly, there was no statistically significant change in the number of motor neuron (MN_*Chat*) nuclei at 26 and 52 weeks or following ASO treatment to 52 weeks (Supplemental Figure 8D and Supplemental Figure 9A), indicating that changes in large-neuron density quantified by unbiased stereology (Figure 4, A and B) reflected soma atrophy rather than neuron loss.

ASO administration did not induce changes in *Ar* mRNA expression in total RNA isolated from the lumbar spinal cord (Figure 4G), consistent with prior results (13). However, motor neurons innervating skeletal muscle constitute only a small portion of the spinal cord cell population, and altered *Ar* expression could have been masked in our analysis of bulk RNA (Supplemental Figure 8D and Supplemental Figure 9A). To address this, we sought to validate whether *Ar* expression in motor neurons and other spinal cord cell types was affected by peripheral ASO treatment. While *Ar* was highly expressed across neuronal populations, there were no discernible effects of peripheral ASO administration on its expression in motor neurons or other spinal cord cell types (Figure 5, C and D). Therefore, this longitudinal snRNA-Seq dataset from AR113Q spinal cord identified major neuronal and glial cell types and indicated that peripheral ASO did not alter the number of motor neuron nuclei or *Ar* expression in spinal cord cells.

### AR113Q spinal cord exhibited age-dependent transcriptional dysregulation.

**Figure 6:**
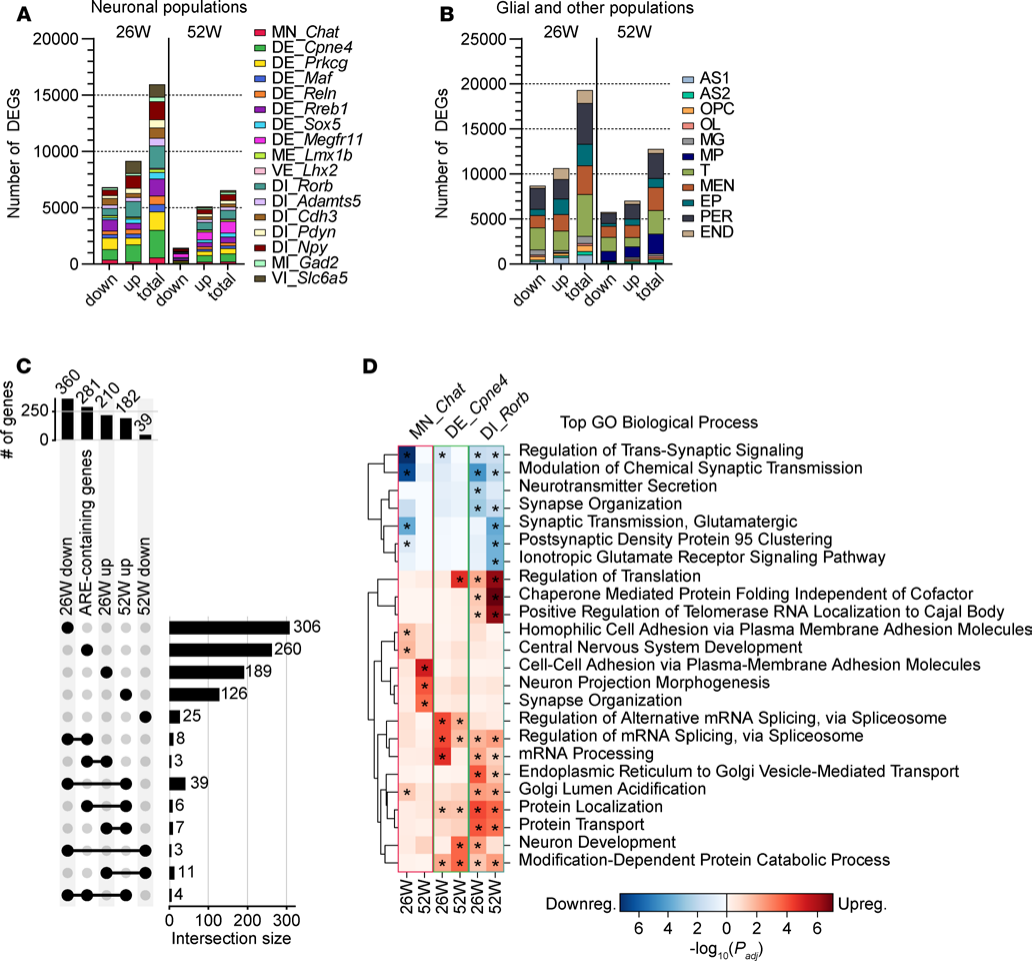
Temporal-specific transcriptional dysregulation in AR113Q spinal cord cells. (**A** and **B**) Number of downregulated, upregulated, and total differentially expressed genes (DEGs; imputed |EMD| ≥ 0.1 and *P*_corrected_ < 0.01) in neurons (**A**) and glia and other populations (**B**) at 26 and 52 weeks. (**C**) UpSet plot showing the number of upregulated and downregulated DEGs in MN_*Chat* at each time point, along with genes containing androgen-responsive element (ARE) as determined by AR-ChIP-Seq (27) and their overlap among gene sets. Vertical bar graphs display total genes in each set, and horizontal bar graphs display unique and overlapping genes with separate and linked dots on each row. (**D**) Heatmap of Gene Ontology (GO) biological process analysis of downregulated (top) and upregulated (bottom) DEGs in MN_*Chat* (left), DE_*Cpne4* (middle), and DI_*Rorb* (right) at 26 and 52 weeks. The top 3 significantly enriched GO terms for each gene set are displayed. Asterisks indicate statistically significant enrichment (adjusted *P* < 0.05).

To investigate the transcriptional effect of the polyQ AR and the impact of peripheral ASO administration, we performed differential gene expression analyses for all cell types identified by snRNA-Seq (Figure 6, A and B, and Supplemental Table 2). Because of the complexity and heterogeneity of gene expression patterns in single-cell transcriptomics, we not only measured fold change in mean expression, but also calculated the earth mover’s distance (EMD) between groups for each gene in every cell type. EMD considers the entire distribution of gene expression levels rather than focusing only on summary statistics, such as means or variances, thereby providing advantages in capturing subtle differences that may reflect biological disturbances in gene expression (24, 25). In the end, this approach was chosen to preserve information pertaining to both the magnitude of change and the proportion of cells undergoing alterations.

We identified differentially expressed genes (DEGs) in each cell type when |EMD| was greater than 0.1 and *P*_corrected_ was less than 0.01. Our analysis (Figure 6, A and B, and Supplemental Table 2) revealed that the sum of all DEGs across cell types declined from 26 to 52 weeks in both neurons and glia. To address whether this apparent decrease was attributable to variations in the number of nuclei among groups, we calculated DEGs from a subsampled dataset, ensuring equal numbers of nuclei for each cell type across all groups. This analysis yielded similar results (Supplemental Figure 10, A and B), indicating that the transcriptional response to polyQ AR in spinal cord was more pronounced in earlier rather than later stages of disease.

Notably, this pattern held true for motor neurons despite the more severe pathology at late stage, as these cells exhibited fewer DEGs at 52 weeks than at 26 weeks (Figure 6C). This raised the question of whether an age-related decline in androgen levels contributed to the reduced number of DEGs observed in 52-week-old mice (26). To address this, we compared DEGs in MN_*Chat* with genes containing androgen-responsive element (ARE) as determined by AR-ChIP-Seq (Figure 6C) (27). Of 281 ARE-containing genes, only 21 overlapped with the upregulated or downregulated MN_*Chat* DEGs at either time point, indicating limited but statistically significant concordance by hypergeometric test (Supplemental Table 3). While the available AR-ChIP-Seq dataset was derived from an analysis of skeletal muscle, where chromatin accessibility may differ from that in spinal cord cells, this comparison suggests that most DEGs in MN_*Chat* were not direct, canonical AR targets.

Interestingly, our analysis also revealed quite limited overlap of DEGs in MN_*Chat* between the 26- and 52-week time points. Only 3 genes overlapped between the downregulated 360 genes at 26 weeks and 39 genes at 52 weeks, while only 7 genes overlapped between the upregulated 210 genes at 26 weeks and 182 genes at 52 weeks (Figure 6C). To delve into the biological implications of the identified transcriptional changes in motor neurons, we performed Gene Ontology (GO) analysis (Supplemental Tables 4 and 5). At 26 weeks, GO terms associated with synaptic regulation were significantly enriched among the downregulated DEGs (Figure 6D). Intriguingly, similar synapse-related terms were upregulated at 52 weeks (Figure 6D), which potentially reflects a compensatory response by motor neurons to counteract non-cell-autonomous damage to NMJs.

In addition to motor neurons, other neuronal populations displayed large numbers of DEGs at both time points (Figure 6A). DE_*Cpne4*, DE_*Prkcg*, DE_*Rreb1*, DI_*Rorb*, and DI_*Npy* were among the neuronal populations that displayed the highest numbers of DEGs at both time points (Figure 6A). Such transcriptional dysregulation in dorsal horn neurons, which process sensory signals (28), may contribute to sensory abnormalities in SBMA patients (4). While numerous DEGs were shared among 2 or more populations of dorsal neurons, each cell type retained a substantial number of unique DEGs (Supplemental Figure 10, C–F). Despite the diverse gene dysregulation among both dorsal excitatory and inhibitory neurons, GO analyses revealed similar downregulated terms related to synaptic function at 26 weeks (Figure 6D, Supplemental Figure 10G, and Supplemental Table 4). A broader array of dysregulated terms was predicted at 52 weeks (Figure 6D and Supplemental Table 5). In addition, VE_*Lhx2*, the most abundant neuronal population in the spinal cord, also exhibited downregulation of synaptic terms at 26 weeks (Supplemental Figure 10G). Unlike MN_*Chat* and dorsal neurons, VE_*Lhx2* did not show dysregulation of synapse-related processes at 52 weeks, suggesting a dynamic transcriptional response among neuronal types that exhibited a shared response at the early disease stage but displayed more divergent dysregulation at the later stage.

### Peripheral ASO administration rescued transcriptional alterations in the spinal cord.

We next sought to investigate the effects of peripheral *AR*-targeted ASO administration on AR113Q spinal cord. While each cell type showed a notable number of DEGs when 52-week ASO-treated AR113Q was compared with 52-week AR113Q or WT, relatively fewer DEGs were observed in comparison with 26-week AR113Q or WT (Supplemental Figure 11, A and B, and Supplemental Table 6). These changes in gene expression between the 52-week groups suggest that peripheral ASO treatment broadly affects the transcriptional landscape in the spinal cord without directly affecting *Ar*.

**Figure 7:**
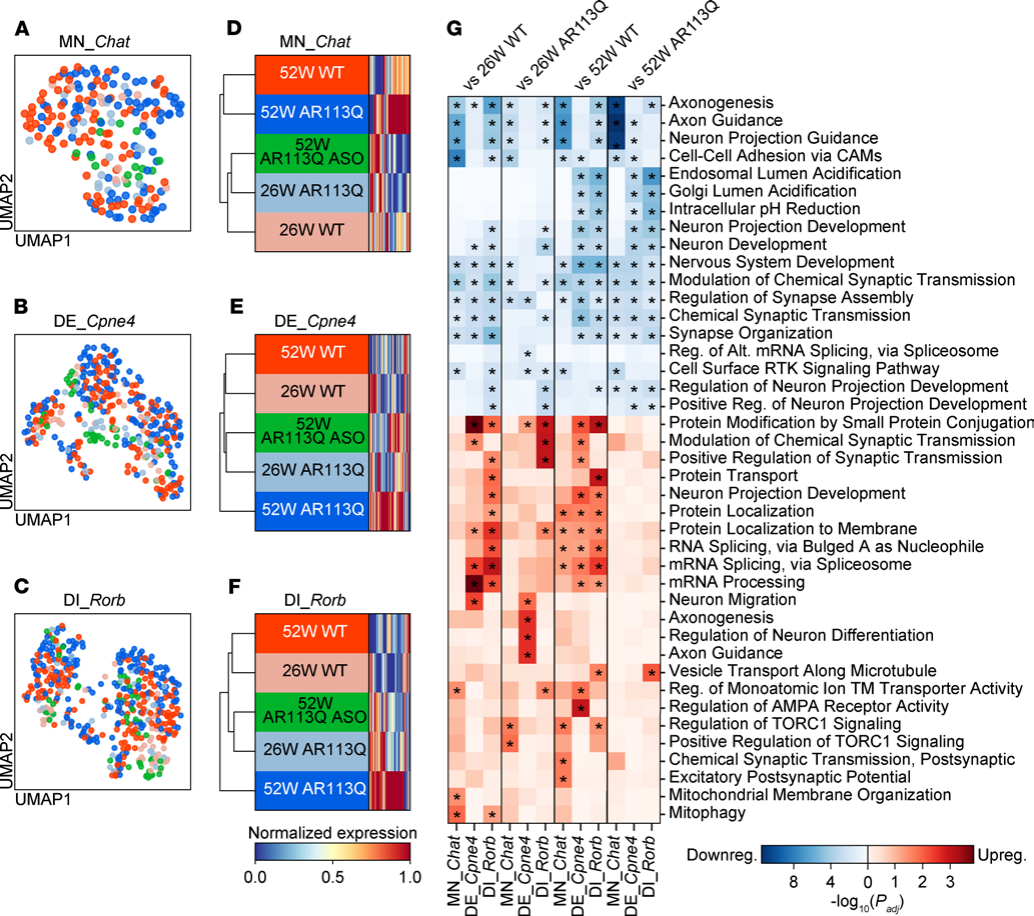
Effect of peripheral *AR*-targeted ASO injection on spinal cord cell types. (**A**–**C**) UMAP embeddings of MN_*Chat* (**A**), DE_*Cpne4* (**B**), and DI_*Rorb* (**C**). (**D**–**F**) Heatmaps with dendrogram showing normalized expression of the respective total DEGs (rows) in MN_*Chat* (**D**), DE_*Cpne4* (**E**), and DI_*Rorb* (**F**) between 52-week AR113Q and 52-week WT. (**G**) Heatmap of GO biological process analysis of downregulated (top) and upregulated (bottom) DEGs in MN_*Chat*, DE_*Cpne4*, and DI_*Rorb* between 52-week AR113Q ASO and other groups. The top 3 significantly enriched GO terms of each gene set are displayed. Asterisks indicate statistically significant enrichment (adjusted *P* < 0.05).

To assess the impact of peripheral ASO administration in an unbiased manner, we performed hierarchical clustering analysis, which calculates the Euclidean transcriptional distance among populations using principal component analysis. Our analysis revealed that ASO-treated 52-week AR113Q cells exhibited more similarity to 26-week AR113Q than to untreated 52-week AR113Q (Figure 7, A–F, and Supplemental Figure 11, C–J). This trend was consistent across all neuronal, glial, and other cell types (Supplemental Figure 12).

Importantly, GO analysis in MN_*Chat* revealed that axon- and synapse-related pathways were further downregulated in ASO-treated AR113Q compared with both 26-week and 52-week untreated AR113Q. This suggests that the rescue of NMJ pathology eliminated the need for compensatory synaptic upregulation, and that the observed downregulation reflects a cell-autonomous effect of persistent mutant AR expression (Figure 7G). Other neurons in ASO-treated AR113Q exhibited less significantly enriched downregulated pathways when compared with 26-week AR113Q than with 52-week AR113Q (Figure 7G, Supplemental Figure 11K, and Supplemental Table 7).

Collectively, these findings suggest that peripheral ASO treatment prevents the progression of transcriptional alterations, such that the transcriptional profile of ASO-treated AR113Q cells at 52 weeks resembles that of untreated AR113Q cells at 26 weeks more closely than it does the untreated AR113Q cells at 52 weeks. Our analyses support a model in which peripheral ASO administration partially rescues skeletal muscle, which non-cell-autonomously mitigates transcriptional alterations and disease progression in spinal cord of aged AR113Q mice.

## Discussion

Our understanding of SBMA pathogenesis builds from observations in mice that degeneration of the neuromuscular system manifests initially in skeletal muscle and progresses to involve spinal motor neurons (7, 8). Here, we sought to test two implications that arise from this model of disease progression: first, that there exists a window of opportunity for therapeutic intervention in symptomatic disease, prior to lower motor neuron degeneration; second, that therapeutic intervention targeting skeletal muscle during this window will arrest the degenerative process affecting the entire neuromuscular system. Our findings support both of these conclusions and provide further experimental evidence to warrant testing of peripherally targeted therapeutics in individuals with SBMA.

These studies enrolled symptomatic AR113Q male mice at 26 weeks of age, a time point at which significant skeletal muscle atrophy and functional weakness are evident. This age was chosen to model an SBMA clinical trial in which individuals with symptomatic disease are enrolled, as we intentionally sought to avoid translational pitfalls that occur with initiation of presymptomatic treatment of mice. At the age of study enrollment, SBMA mice had no morphologically detectable NMJ or lower motor neuron pathology, despite the occurrence of skeletal muscle atrophy and diminished motor function. We note that our analysis of NMJ pathology focused on the colocalization of pre- and postsynaptic markers as an assessment of denervation, whereas others have shown changes at this time point that may precede frank denervation (29). Disease progression occurred over the ensuing 6 months, and at the study’s endpoint at 52 weeks mutant males exhibited significant muscle atrophy accompanied by NMJ denervation and lower motor neuron soma atrophy. All of these pathologies were improved by peripheral administration of *AR*-targeted ASO, which also rescued survival and resulted in the significant knockdown of *Ar* gene expression in skeletal muscle but not spinal cord. It is notable that the therapeutic benefits reported here occurred with approximately 50% knockdown of *Ar* mRNA in muscle, a finding that mirrors the threshold for therapeutic benefits in younger SBMA mice that were treated for a shorter duration (13). Whether the therapeutic threshold in SBMA patients is similar is currently unknown, but the beneficial effects shown in mice with modest gene knockdown are encouraging. Notably, while peripheral ASO administration rescued many disease manifestations, neither grip strength nor body weight was significantly improved. As ASO treatment rescues gain-of-function mechanisms while exacerbating loss-of-function, the observed limitations of this therapeutic approach may reflect an exacerbated loss of AR’s anabolic support. Other possible limitations associated with peripheral ASO administration cannot be excluded, including the intrinsic vulnerability of spinal motor neurons to polyQ AR toxicity. Together, these factors limited recovery of grip strength despite beneficial effects on muscle fiber size and survival.

We performed snRNA-Seq on the lumbar spinal cord to unbiasedly identify cell type– and age-dependent gene expression changes in AR113Q males and to characterize the response to peripherally administered ASO. Several notable conclusions emerged from this analysis. First, endogenous polyQ AR is broadly expressed across neuronal cell types but is expressed at much lower levels in glial cells of the spinal cord. Its expression in dorsal spinal neurons is particularly intriguing given the sensory neuropathy that occurs in individuals with SBMA (4) and is an area that warrants future investigation. Second, gene expression changes are detectable in all cell types and precede the occurrence of morphologically detectable NMJ and lower motor neuron pathology. Third, as AR113Q mice age from 26 to 52 weeks, the number of DEGs in the spinal cord decreases, potentially reflecting the activation of compensatory responses. This pattern is reminiscent of observations in the cerebellum of spinocerebellar ataxia type 1 mouse models (25). Fourth and perhaps most strikingly, peripherally administered ASO has unanticipated effects on spinal cord gene expression, even though this intervention does not alter the expression of *Ar* mRNA in any spinal cord cell type. These effects are seen across all cell types, resulting in gene expression patterns in 52-week ASO-treated AR113Q males that most closely resemble those of 26-week AR113Q spinal cords. These findings indicate that peripherally targeted polyQ AR knockdown has broad beneficial effects in resetting the AR113Q spinal cord transcriptional landscape.

In summary, our study demonstrates the effectiveness of peripheral *AR*-targeted ASO in rescuing both skeletal muscle and spinal cord defects in SBMA mice, even after the onset of symptoms. While several strategies to mitigate polyQ AR toxicity are under investigation (30), our findings highlight the therapeutic benefits of targeting peripheral *AR* gene expression, even in the symptomatic stages of disease. The age-dependent transcriptional dysregulation and response within the spinal cord to peripheral ASO treatment highlight the complexity of SBMA pathogenesis and the promise of systemic interventions. Our findings offer valuable insights into the dynamic interplay between peripheral tissues and the central nervous system in SBMA, paving the way for further research and potential clinical applications to the treatment of neuromuscular disorders.

## Methods

Further information can be found in Supplemental Methods.

### Sex as a biological variable

As SBMA is an X-linked, sex-limited disorder that affects only males with the mutant allele, only male mice were used in this study.

### Antibodies and related reagents

#### Primary antibodies.

The following primary antibodies (antigen, dilution, vendor, catalog number) were used for these studies: neurofilament, 1:500, Abcam, ab8135; synaptophysin, 1:100, MilliporeSigma, ZRB1317; myelin basic protein (MBP), 1:200, Abcam, ab7349; NeuN, 1:200, Abcam, ab177487; choline acetyltransferase, 1:100, MilliporeSigma, AB144P.

#### Secondary antibodies.

The following secondary antibodies (antigen, dilution, vendor, catalog number) were used: Alexa Fluor 488–conjugated donkey anti-rat antibody, 1:400, Thermo Fisher Scientific, A-21208; Alexa Fluor 594–conjugated donkey anti-rabbit antibody, 1:400, Thermo Fisher Scientific, A-21207; Alexa Fluor 594–conjugated donkey anti-goat antibody, 1:400, Thermo Fisher Scientific, A-11058.

#### Other fluorescent probes.

We also used Alexa Fluor 594–conjugated α-bungarotoxin, 1:100, Thermo Fisher Scientific, B13423 and Alexa Fluor 488–conjugated wheat germ agglutinin, 10 μg/mL, Thermo Fisher Scientific, W11261.

### Mice

AR113Q mice were generated using gene targeting to insert human *AR* sequence with an expanded CAG repeat into mouse *Ar* exon 1, as previously described (9, 10). Animals were backcrossed to C57BL/6J for greater than 10 generations. Mice were housed in a specific pathogen–free facility and maintained on a 12-hour light/12-hour dark cycle with chow and water ad libitum. Genotypes were verified by PCR on ear samples obtained 3 weeks after birth, using the forward primer 5′-CCAGAATCTGTTCCAGAGCGTG-3′ (MilliporeSigma, 6-FAM labeled) and the reverse primer 5′-TGTTCCCCTGGACTCAGATG-3′ (Invitrogen). CAG repeat length of all AR113Q males used in this study was assessed by fragment analysis PCR (Laragen). C57BL/6J mice were from The Jackson Laboratory (strain 000664). Survival was assessed by humane endpoints including >20% loss of maximal body weight or identification of moribund behavior.

### ASO treatment

ASOs were made of a 16-mer, 4-8-4 constrained mixed cEt/methoxyethyl (MOE) gapmer with full phosphorothioate backbone modification containing the following sequence: AAGTTGTAGTAGTCGC, which is complementary to human and mouse *AR* transcripts as detailed previously (13). In these ASOs, cEt sugar modifications were present at positions 1, 3, 14, and 16, while 2′ MOE sugar modifications were present at positions 2, 4, 13, and 15. This ASO is designated ASO1 in Supplemental Figure 2. Alternatively, ASO1 conjugated to palmitic acid via a phosphodiester linkage at the 5′ end is designated ASO2 in Supplemental Figure 2. Non-targeted ASO, with the same chemistry as ASO2, contained the following sequence: CGCTATACTAATCATAT. ASOs were diluted in PBS, pH 7, and administered subcutaneously to C57BL/6J males (5–100 mg/kg) or AR113Q males (25 mg/kg) weekly for the indicated durations. AR113Q mice were given ASO2 or vehicle (PBS, pH 7) for the treatment study. For this longitudinal trial, AR113Q males were randomly assigned to vehicle or ASO treatment group. The CAG repeat range for the vehicle group ranged from 97 to 111 repeats with a mean of 105; the CAG repeat range for the treatment group ranged from 95 to 110 repeats with a mean of 104 (*P* > 0.05 by Student’s *t* test).

### Tissue preparation

Mice were perfused with pre-cooled 4% paraformaldehyde (PFA). The spinal cord (lumbar enlargement) was removed from the vertebral canal by laminectomy and postfixed in 4% PFA for another 24 hours at 4°C, then embedded in optimal cutting temperature (OCT) compound and frozen in pre-chilled isopentane. TA muscle was harvested from non-perfused mice, embedded in OCT, and frozen in pre-chilled isopentane. Tissues were stored at –80°C until sectioning.

### Immunohistochemistry

Slides were fixed in ice-cold methanol for 5 minutes. For muscle fiber quantification, slides were incubated in 10 μg/mL Alexa Fluor 488–conjugated wheat germ agglutinin (abbreviated FITC-WGA) for 10 minutes at room temperature. For spinal cord, slides were blocked for 1 hour at room temperature with 5% normal donkey serum in PBS containing 0.1% Triton X (PBS-T), followed by incubation with primary antibody in blocking buffer overnight at 4°C. After washes, slides were incubated with secondary antibody in blocking buffer for 1 hour at room temperature. Slides were mounted using antifade mounting medium with DAPI (Vector Laboratories). Images were captured on a Nikon A1R confocal microscope or Zeiss Axio Imager Z1 microscope.

### Neuron quantification

The spinal lumbar enlargement was serially sectioned in 20 μm transverse sections through its entirety, with each section mounted sequentially on slides. For each mouse, one slide near the rostral end of the lumbar enlargement was selected, and every tenth slide was subsequently selected through the caudal end of the specimen. Slides were analyzed by immunofluorescence staining for NeuN and MBP. Digital images were acquired by a Zeiss Axio Imager Z1 microscope with an automated stage. Images were further cropped to the ventral gray matter. Processed images were analyzed by an automated CellProfiler pipeline (https://cellprofiler.org/), and neurons with a soma diameter greater than 30 μm were quantified.

### Muscle fiber quantification

TA muscle was cut in cross section at a thickness of 5 μm. Muscle fibers were visualized by staining with FITC-WGA. Digital images were captured using a Zeiss Axio Imager Z1 microscope with an automated stage. The area of each muscle fiber was quantified using an automated CellProfiler pipeline. Approximately 100 adjacent fibers from each section were measured.

### NMJ quantification

TA muscle was cut longitudinally at a thickness of 30 μm. NMJs were visualized using staining with Alexa Fluor 594–conjugated α-bungarotoxin (motor endplates) and with antibodies against neurofilament (axon terminals) and synaptophysin (synaptic vesicles). Digital images were captured using a Zeiss Axio Imager Z1 microscope with an automated stage. Colocalization between pre- and postsynaptic markers was measured by automated CellProfiler pipeline in 100 NMJs per mouse. NMJs were binned according to colocalization as follows: fully innervated, >50%; partially innervated, <50%, >20%; denervated, <20%.

### Grip strength

The grip strength meter (Columbus Instruments) was positioned horizontally. Mice were allowed to grasp the pull bar with their forelimbs, and then were pulled backward in the horizontal plane. The force was recorded at the moment the pull bar was released. The test was repeated 5 consecutive times per session, and the highest value from the 5 trials was recorded as the grip strength.

### Quantitative reverse transcriptase PCR

RNA was isolated from mouse tissues at the indicated time points using TRIzol (Thermo Fisher Scientific) extraction according to the manufacturer’s instructions. RNA was reverse-transcribed using the High Capacity cDNA Reverse Transcription Kit (Applied Biosystems, 4368814). Quantitative reverse transcriptase PCR was performed using FastStart TaqMan Probe Master Mix (Roche, 4673409001), an ABI7900HT Sequence Detection System (Applied Biosystems), and gene-specific FAM-labeled TaqMan primer/probe mix for *Ar* (Mm00442688_m1, Thermo Fisher Scientific). Gene expression was normalized to *Cpsf2*-Vic (Mm00489754_m1, Thermo Fisher Scientific) multiplexed within the same well. Relative expression was calculated by the standard curve method.

### Isolation of nuclei from frozen tissue

Isolation of nuclei from frozen tissue followed a previously established protocol (31). Lumbar cords from 3 animals per genotype (WT and AR113Q) at each time point (26 and 52 weeks) were used, with 6 animals for the ASO-injected 52-week AR113Q group. Briefly, frozen tissue underwent gentle Dounce homogenization in 2 mL of ice-cold Nuclei EZ Prep buffer (MilliporeSigma, NUC101-1KT) using a large clearance pestle “A” followed by a small clearance pestle “B,” each for 25 strokes. Subsequently, the homogenized tissue was incubated on ice for 5 minutes with an additional 2 mL of cold EZ Prep buffer. The homogenates were then centrifuged at 500*g* for 5 minutes at 4°C. The nucleus pellets were resuspended in 4 mL of cold EZ Prep buffer, followed by incubation on ice for 5 minutes and a repeated centrifugation at 500*g* for 5 minutes at 4°C. The nuclei were washed in 4 mL of nuclei suspension buffer (NSB), which contained 1× PBS, 0.01% BSA, and 0.1% RNase inhibitor (Clontech/Takara, 2313B). The final washing step was followed by centrifugation at 500*g* for 5 minutes at 4°C. The purified nuclei were resuspended in 1 mL of NSB, filtered through a 40 μm cell strainer (Fisher Scientific, 22-363-547), and quantified using a Countess III FL Cell Counter (Thermo Fisher Scientific, AMQAF2000). Single-nucleus suspensions were then appropriately diluted to achieve a concentration of approximately 1,000 nuclei per microliter in NSB for library preparation.

### 10x Genomics snRNA-Seq

Libraries were prepared from diluted single-nucleus suspensions using 10x Genomics Chromium Single Cell 3′ Reagent Kits v3.1 at the Yale Center for Genome Analysis (YCGA). Briefly, 10,000 cells per sample were mixed with RT Master Mix, loaded onto the Single Cell A Chip, and combined with approximately 750,000 barcoded gel beads to form nanoliter-scale Gel Beads-In-Emulsion (GEMs). Each gel bead had primers containing the following: an Illumina R1 sequence (read 1 sequencing primer), a 16-nucleotide barcode, a 12-nucleotide unique molecular identifier (UMI), and a 30-nucleotide poly-dT primer sequence. After dissolution of the gel beads in a GEM, the released primers were mixed with the cell lysate and Master Mix, resulting in barcoded, full-length cDNA from polyadenylated mRNA after incubation. Silane magnetic beads were then used to remove leftover biochemical reagents from the post-GEM reaction mixture.

Subsequently, full-length, barcoded cDNA underwent PCR amplification to generate sufficient mass for library construction. Enzymatic fragmentation and size selection were used to optimize the cDNA amplicon size before library construction. During library construction, P5, P7, a sample index, and R2 (read 2 primer sequence) were added via end repair, A-tailing, adapter ligation, and PCR. The final libraries contained the P5 and P7 primers used in Illumina bridge amplification. Sequencing was performed using the Illumina NovaSeq6000 at the YCGA.

Sample concentrations were normalized to 1.2 nM and loaded onto an Illumina NovaSeq S4 flow cell at a concentration that yielded 20,000–50,000 passing filter clusters per cell per sample. Paired-end sequencing was conducted on an Illumina NovaSeq6000 instrument following Illumina protocols and 10x sequencing specifications. The 8 bp index was read during an additional sequencing read that automatically followed the completion of read 1. Data generated during sequencing runs were simultaneously transferred to the YCGA high-performance computing cluster. To monitor quality in real time, a positive control (prepared bacteriophage PhiX library) provided by Illumina was spiked into every lane at a concentration of 0.3%. Signal intensities were converted to individual base calls during a run using the system’s Real Time Analysis software. Base calls were transferred from the machine’s dedicated personal computer to the Yale high-performance computing cluster via a 1-gigabit network mount for downstream analysis.

### Quantification and statistical analysis

#### Statistics.

Statistical analysis was performed in GraphPad Prism 9.0 using 2-tailed unpaired Student’s *t* test for comparisons of 2 groups and 1-way ANOVA with Tukey’s multiple-comparison test for groups of 3 or more. The log-rank test with Bonferroni’s correction was used for comparisons of survival across multiple groups. α < 0.05 was set as the threshold for significance.

#### Integration and clustering of snRNA-Seq data.

Following alignment of sequencing reads to the pre-mRNA–containing mouse reference genomes (mm10) using the CellRanger v7.1.0 count function (10x Genomics), ambient RNA was removed using CellBender v0.3.0 (32). It is noteworthy that cDNA fragments (reads) from human *AR* exon 1 and the CAG tract expansion targeted to mouse *Ar* exon 1 in AR113Q mice may have not accurately aligned to the mouse genome reference (mm10), potentially resulting in a slight reduction in *Ar* read counts in AR113Q mice. We followed the standard scRNA-Seq analysis pipelines for pre-processing scRNA-Seq count data (33–35). All snRNA-Seq data were analyzed with Python (version 3.8.2), and pre-processing was performed using SCANPY v1.6.0 (36). Briefly, we removed genes that were expressed in fewer than 3 nuclei and removed nuclei that expressed fewer than 500 genes before imputation, as well as nuclei in which more than 5% of the total counts aligned to mitochondrial genes. The resulting UMI counts were normalized to library size and square-root-transformed. Doublet detection was performed using Scrublet (37), and identified doublets were removed.

To integrate snRNA-Seq, we used an approximate batch-balanced, k-nearest neighbors (BBKNN) graph for batch-effect correction, manifold learning, and clustering (38). We used SCANPY’s fast approximation implementation of BBKNN with 100 principal components to calculate pairwise Euclidean distances but otherwise default parameters (36, 39). For each cell, the 3 nearest neighboring cells in each condition were identified by Euclidean distance in 100-dimensional principal component analysis space. This k-nearest neighbors (KNN) graph was used as the basis for downstream analysis.

To visualize the snRNA-Seq data, we implemented various non-linear dimension reduction methods with the BBKNN batch-corrected connectivity matrix as input for uniform manifold approximation and projection (UMAP) (40, 41). For clustering nuclei, we used the Leiden community detection method applied to the BBKNN graph with high resolution (resolution = 3, otherwise default parameters using SCANPY’s implementation), which facilitated cell type annotation. Then, pre-clusters of the same major spinal cord type were merged when appropriate and manually annotated based on expression of previously described cell type–specific marker genes (23). Pre-clusters expressing marker genes of multiple cell types or lacking marker gene expression were removed. Unless otherwise noted, these merged clusters, rather than individual pre-/subclusters, were used for downstream analyses.

#### Differential expression and Gene Ontology analysis.

Markov affinity-based graph imputation of cells (MAGIC) was used for gene expression imputation based on the BBKNN graph (42). Differentially expressed genes (DEGs) were calculated as previously described (25). Briefly, to identify global gene expression changes across a population of cells and to detect complex variations arising from heterogeneous changes in subsets of cells or alterations in population subcluster composition (43), we used a combination of 3 metrics to determine DEGs: the Wasserstein or earth mover’s distance (EMD), an adjusted *P* value from a 2-sided Mann-Whitney *U*/Wilcoxon’s rank-sum test with a continuity correction and a *P* value adjustment using the Benjamini-Hochberg correction, and the binary logarithm of fold change between mean counts. The EMD, representing the minimal cost to transform one distribution to another, has been used to assess gene expression that significantly differs between conditions (44, 45). Binary comparisons of genotype were performed for each time point, treatment condition, and cell type for imputed expression (batch effect corrected and conditioned on genotype) and for normalized and transformed raw data (data not shown). For subsampling to address differences in the number of nuclei across groups, each cell type’s count across groups was adjusted to match the count of the group with the fewest nuclei for that cell type. This ensured an equal number of nuclei for each cell type across all groups. Genes with *P*_corrected_ < 0.01 and |EMD| ≥ 0.1 were considered significant. Gene set enrichment analyses of significantly differentially expressed genes were performed through overrepresentation analysis using Enrichr (46, 47) and GSEApy (48). Terms of GO Biological Process 2025 were considered significant below a threshold of adjusted *P* < 0.05.

### Study approval

All procedures involving mice were approved by the University of Michigan Committee on Use and Care of Animals (PRO00010017) and conducted in accordance with institutional and federal guidelines.

### Data and material availability

Supporting data values are provided in the Supporting Data Values file. All sequencing data are available from the NCBI’s Gene Expression Omnibus database (GEO GSE264689 snRNA-Seq, GSE300077 bulk RNA-Seq; Supplemental Figure 5). Scripts used to analyze the snRNA-Seq data are available at https://github.com/ChrLeeee/AR113Q-snRNA-Seq for academic use (commit ID: 4fcd1af9ec685b69c5bf33cf8b0f6f4260e158aa) (https://github.com/ChrLeeee/AR113Q-snRNA-seq/commit/4fcd1af9ec685b69c5bf33cf8b0f6f4260e158aa).

Additional data are available upon reasonable request.

## Author contributions

JL and APL conceptualized the study. CL, ZY, CJK, LT, RMG, and EB performed investigation. JL and APL acquired funding. JL and APL supervised the study. HTZ provided unique reagents. CL, ZY, CJK, JL, and APL analyzed data. CL, JL, and APL wrote the manuscript. CL, ZY, CJK, LT, RMG, EB, HTZ, JL, and APL reviewed and edited the manuscript.

## Funding support

This work is the result of NIH funding, in whole or in part, and is subject to the NIH Public Access Policy. Through acceptance of this federal funding, the NIH has been given a right to make the work publicly available in PubMed Central.

- NIH R01NS119873 to APL.
- NIH R01AG076154 to JL.
- NIH T32GM145470 and T32GM007863 to CJK.

## Supplementary Material

Supplemental data

Unedited blot and gel images

Supplemental table 1

Supplemental table 2

Supplemental table 3

Supplemental table 4

Supplemental table 5

Supplemental table 6

Supplemental table 7

Supporting data values

## Version 1 08/28/2025

In-Press Preview

## Version 2 11/03/2025

Electronic publication
