# Supplementary material for: Peripherally administered androgen receptor–targeted antisense oligonucleotide rescues spinal pathology in a murine SBMA model
Source: J Clin Invest. 2025 Aug 28;135(21):e182955. doi: 10.1172/JCI182955 (PMC12578385; doi:10.1172/JCI182955)
Supplement: Supplemental data [file jci-135-182955-s008.pdf]

Supplemental Material

Peripherally administered androgen receptor-targeted antisense oligonucleotide rescues spinal pathology in a murine SBMA model

Supplemental Methods

Western blot

Tissue was minced and homogenized in RIPA Lysis and Extraction Buffer (G-Biosciences; 786-490) with cOmplete Mini protease inhibitor (Roche; 46264500) and centrifuged at 15,000 x *g* for 15 minutes. Soluble protein in the supernatant was quantified using DC-Assay (BioRad; 5000112). Gels were run with equal amounts of protein in each well in a NuPAGE Bis-Tris Mini Protein Gel, 4%-12% (Invitrogen; NP0335BOX) for 1.5-2 hours at 130 V in 1x NuPAGE MOPS SDS running buffer (Invitrogen; NP0001). Protein was transferred to a PVDF membrane (Merck Millipore; IPVH00010) for 2 hours at 13 V in a semi-dry transfer apparatus, which was then blocked in 5% milk at room temperature for 30-60 minutes and incubated in primary antibody at 4°C overnight. Membranes were incubated in secondary antibody for 1 hour at room temperature. Protein was visualized using either Pierce ECL Western Blotting Substrate (Thermo Fisher Scientific; 32106) or SuperSignal West Pico PLUS Chemiluminescent Substrate (Thermo Fisher Scientific; 34577). Imaging was performed with an iBright FL1500 system (Invitrogen; A44241). Band intensity was quantified with Fiji (1), with membrane background subtracted, and bands were normalized to the indicated loading control.

Antibodies and related reagents

Primary antibodies. The following primary antibodies (antigen, dilution/concentration, vendor, cat. no.) were used for these studies: Anti-Androgen

Receptor PG-21, 1:500, EMD Millipore, 06-680; Beta Actin AC-15, 1:2,000, Invitrogen, MA1-913999; Vinculin, 1:10,000, Sigma Aldrich, V9131.

Secondary antibodies. The following secondary antibodies (antigen, dilution/concentration, vendor, cat. no.) were used: Goat anti-mouse IgG (H+L)-HRP conjugate, 1:2,000, Bio-Rad, 170-6516; Goat anti-rabbit IgG (H+L)-HRP conjugate, 1:2,000, Bio-Rad, 170-6515.

Other. WGA-Alexa Fluor Plus 405, 5 µg/mL, Thermo Fisher, W56132.

Bulk RNA-sequencing

RNA was isolated from flash-frozen lumbar spinal cord by use of a phenol/chloroform extraction protocol. Briefly, tissue was minced with surgical scissors and homogenized with an OMNI homogenizer in TRIzol (Invitrogen; 15596-026), with total RNA then extracted with chloroform and isopropanol. 500 ng total RNA per sample, measured by NanoDrop, was used for sequencing. Library prep and next-generation sequencing were carried out by the Advanced Genomics Core at the University of Michigan. The pool was subjected to 150 bp paired-end sequencing according to the manufacturer's protocol (Illumina NovaSeqXPlus, System Suite Version: 1.3.0.39308). BCL Convert Conversion Software v4.3.13 (Illumina) was used to generate de-multiplexed fastq files, which were then input into the NF-Core RNA-seq pipeline (2) for quality control, trimming, alignment, and quantification by star-rsem. Full software version numbers used are included below.

Following gene expression quantification, the resulting expected count matrix was then input into DESeq2 (3) for differential expression analysis by R v4.4.3, and thresholds of p-adj < 0.05 (as determined by the Wald test with Benjamini-Hochberg correction) and

$|log_2FC| > 1$ were used for statistical and biological magnitude significance, respectively. Prism 10 (GraphPad) was used for data visualization.

Data analysis

The following software versions were used by the NF-Core RNA-seq pipeline for data processing of the bulk RNA-sequencing in Supplemental Figure 5.

BEDTOOLS_GENOMECOV_FW:	bedtools: 2.31.1
CUSTOM_GETCHROMSIZES:	getchromsizes: 1.2
DUPRADAR:	bioconductor-dupradar: 1.32.0
FASTQC:	fastqc: 0.12.1
FQ_SUBSAMPLE:	fq: 0.9.1 (2022-02-22)
GTF2BED:	perl: 5.26.2
GTF_FILTER:	python: 3.9.5
MULTIQC_CUSTOM_BIOTYPE:	python: 3.9.5
PICARD_MARKDUPLICATES:	picard: 3.1.1
QUALIMAP_RNASEQ:	qualimap: 2.3
RSEM_CALCULATEEXPRESSION:	rsem: 1.3.1
	star: 2.7.10a
RSEM_MERGE_COUNTS:	sed: 4.7
RSEQC_BAMSTAT:	rseqc: 5.0.2
RSEQC_INFEREXPERIMENT:	rseqc: 5.0.2
RSEQC_INNERDISTANCE:	rseqc: 5.0.2
RSEQC_JUNCTIONANNOTATION:	rseqc: 5.0.2
RSEQC_JUNCTIONSATURATION:	rseqc: 5.0.2
RSEQC_READDISTRIBUTION:	rseqc: 5.0.2

RSEQC_READDUPLICATION:	rseqc: 5.0.2
SALMON_QUANT:	salmon: 1.10.1
SAMTOOLS_FLAGSTAT:	samtools: 1.2
SAMTOOLS_IDXSTATS:	samtools: 1.2
SAMTOOLS_INDEX:	samtools: 1.2
SAMTOOLS_SORT:	samtools: 1.2
SAMTOOLS_STATS:	samtools: 1.2
STRINGTIE_STRINGTIE:	stringtie: 2.2.1
SUBREAD_FEATURECOUNTS:	subread: 2.0.1
TRIMGALORE:	trimgalore: 0.6.7
	cutadapt: 3.4
UCSC_BEDCLIP:	ucsc: 377
UCSC_BEDGRAPHTOBIGWIG:	ucsc: 445
Workflow:	nf-core/rnaseq: v3.16.1-g1f3f64d
	Nextflow: 24.10.5

Supplemental References

1. Schindelin J, Arganda-Carreras I, Frise E, Kaynig V, Longair M, Pietzsch T, et al. Fiji: an open-source platform for biological image analysis. *Nat Methods.* 2012;9(7):676-682.
2. Ewels P, Peltzer A, Fillinger S, Patel H, Alneberg J, Wilm A, et al. The nf-core framework for community-curated bioinformatics pipelines. *Nat Biotech.* 2020;38:276-278.
3. Love MI, Huber W, Anders S. Moderated estimation of fold change and dispersion for RNA-seq data within DESeq2. *Genome Biol.* 2014;15:550.

Supplemental Tables

Supplemental Table 1. Sample information for snRNA-seq analysis.

The table lists the sample ID, age, and genotype of animals used in the snRNA-seq analysis, along with the number of nuclei per cell type in each animal.

Supplemental Table 2. Imputed differential gene expression between WT and AR113Q at each timepoint, and among WT, AR113Q and AR113Q ASO.

The table contains lists of upregulated and downregulated genes for each cell type after imputation within groups. Earth Mover's Distance (EMD) and log2(fold-change) were calculated to compare gene expression distributions. Genes with |EMD| > 0.1 and Benjamini-Hochberg adjusted P-value ($P_{corrected}$) <0.01 were considered significant. The data is organized by sheet.

Supplemental Table 3. Hypergeometric test of ARE-containing genes and the MN_*Chat* DEGs.

The table presents the results of hypergeometric tests assessing the overlap between the MN_*Chat* DEGs at 26 and 52 weeks and ARE-containing genes identified by AR-ChIP-seq.

Supplemental Table 4. Gene ontology of differentially expressed genes between WT and AR113Q at 26 weeks.

The table contains the results of GO analyses (Biological Process 2025) for upregulated and downregulated DEGs between AR113Q and WT at 26 weeks. Significance was determined based on P-values (*adjusted P-value*<0.01). The data is organized by sheet.

Supplemental Table 5. Gene ontology of differentially expressed genes between WT and AR113Q at 52 weeks.

The table contains the results of GO analyses (Biological Process 2025) for upregulated and down-regulated DEGs between AR113Q and WT at 52 weeks. Significance was determined based on P-values (*adjusted P-value*<0.01). The data is organized by sheet.

Supplemental Table 6. Gene ontology of differentially expressed gene between AR113Q ASO and WT at 26 and 52 weeks.

The table contains the results of GO analyses (Biological Process 2025) for upregulated and down-regulated DEGs between AR113Q ASO and WT at 26 and 52 weeks. Significance was determined based on P-values (*adjusted P-value*<0.01). The data is organized by sheet.

Supplemental Table 7. Gene ontology of differentially expressed genes between AR113Q ASO and AR113Q at 26 and 52 weeks.

The table contains the results of GO analyses (Biological Process 2025) for up- and down-regulated DEGs between AR113Q ASO and AR113Q at 26 and 52 weeks. Significance was determined based on P-values (*adjusted P-value*<0.01). The data is organized by sheet.

Supplemental Figures

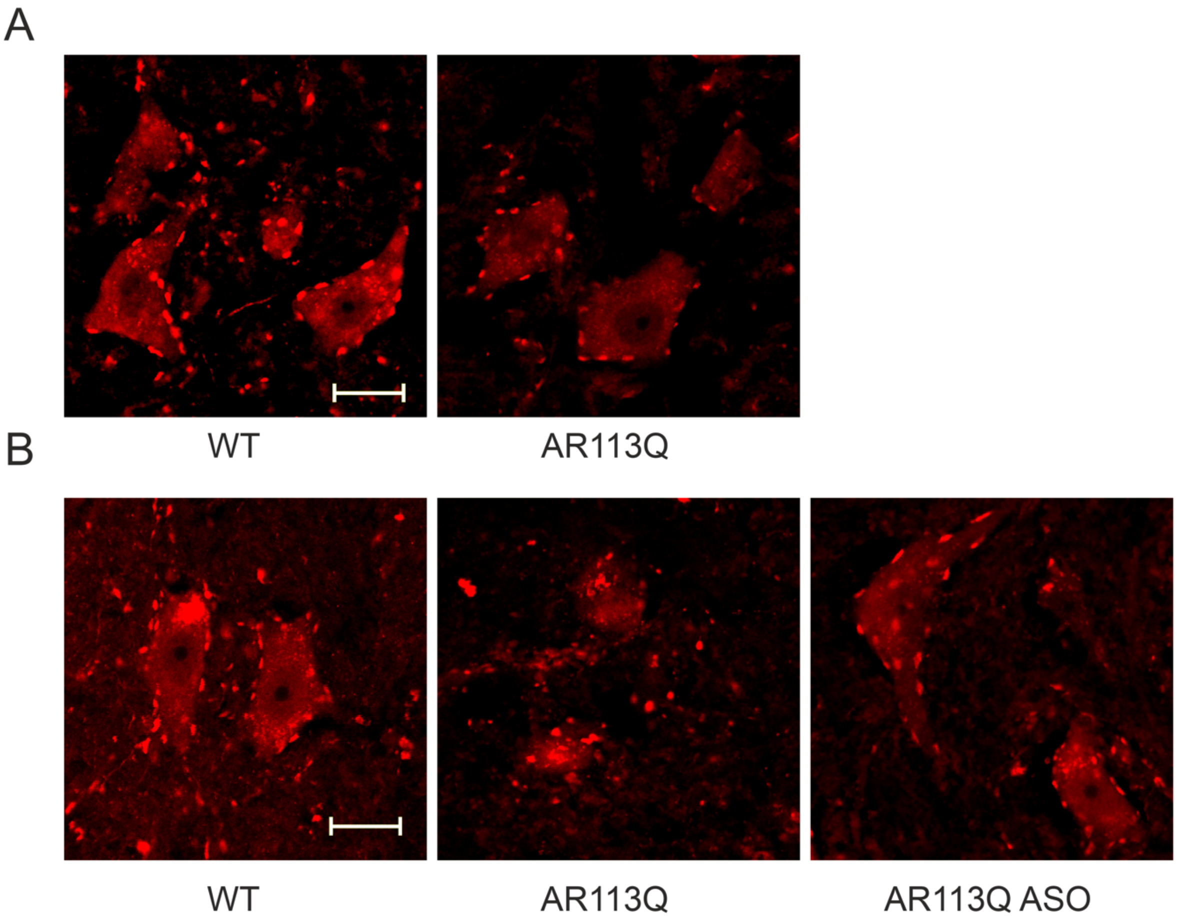

Supplemental Figure 1. Choline acetyltransferase-positive neurons in ventral lumbar enlargement.

(A) 26-week WT and AR113 males; (B) 52-week WT, AR113Q, or AR113Q + ASO. ChAT in red. Scale bar = 25 μm.

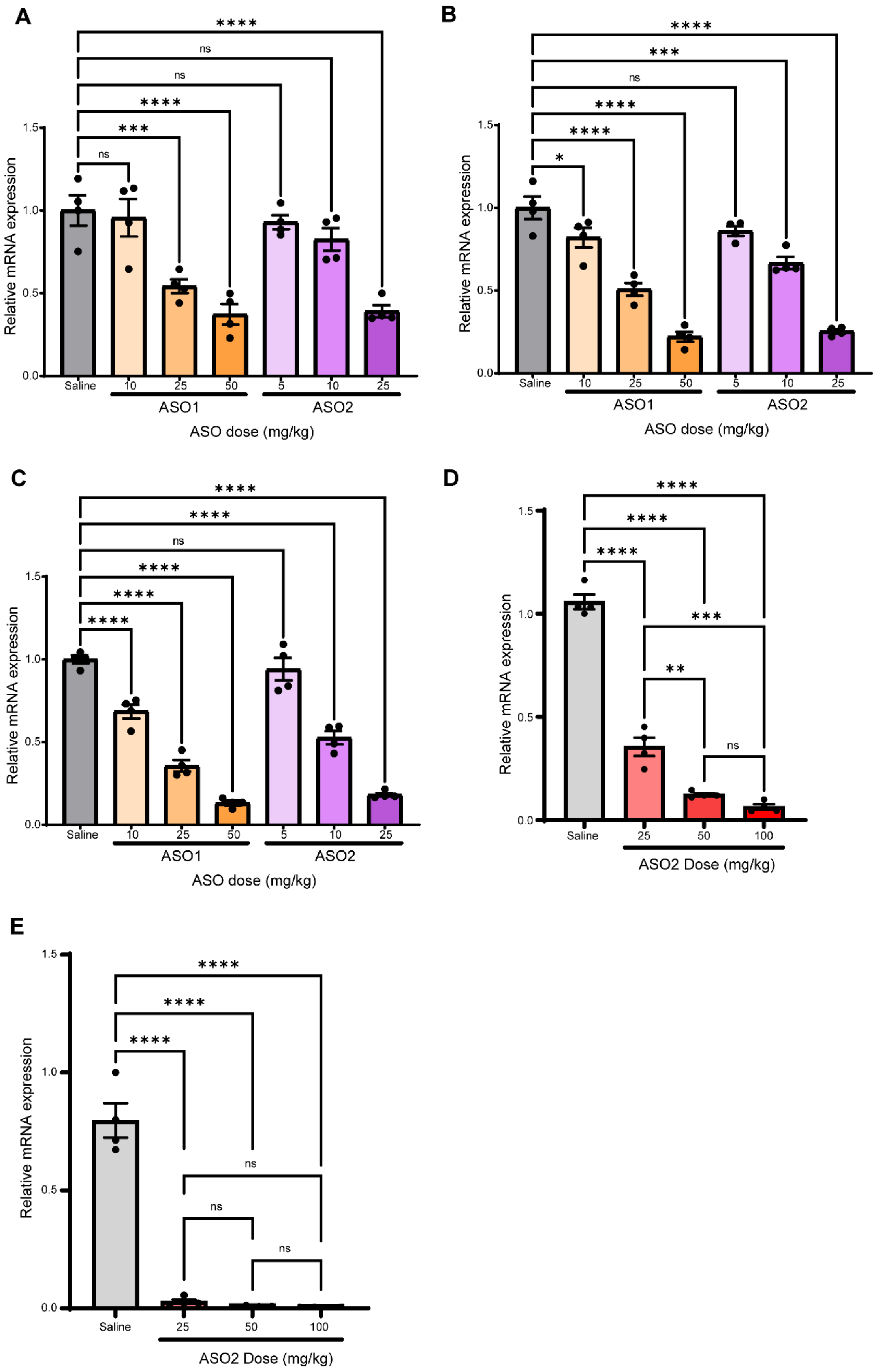

A
B
C
D
E
Relative mRNA expression
0.0
0.5
1.0
1.5
Saline
10
25
50
5
ASO1
ASO2
ASO dose (mg/kg)
100
ASO2 Dose (mg/kg)
ns
*
**
***
****

Supplemental Figure 2. Dose-dependent knockdown of *Ar* mRNA in skeletal muscle and liver by ASO.

A.–C. C57BL/6J males (4 mice/group) were administered ASO or vehicle subcutaneously, once per week for 4 weeks starting at 8 weeks of age. Relative *Ar* mRNA levels by qPCR were determined in (A) TA muscle (mean ± s.e.m., ns, not significant, *$p < 0.05$, ***$p < 0.001$, ****$p < 0.0001$ by one-way ANOVA with Tukey's multiple comparisons test, $F = 15.16$, $df = 6$), (B) soleus (mean ± s.e.m., ns, not significant, ***$p < 0.001$, ****$p < 0.0001$ by one-way ANOVA with Tukey's multiple comparisons test, $F = 49.49$, $df = 6$), and (C) diaphragm (mean ± s.e.m., ns, not significant, *$p < 0.05$, ****$p < 0.0001$ by one-way ANOVA with Tukey's multiple comparisons test, $F = 84.13$, $df = 6$).

D., E. C57BL/6J males (4 mice/group) were administered ASO2 (25, 50, or 100 mg/kg) or vehicle subcutaneously, once per week for 4 weeks starting at 8 weeks of age. Relative *Ar* mRNA levels by qPCR were determined in TA muscle (D) and liver (E). Data are mean ± s.e.m. ns, not significant, **$p < 0.01$, ***$p < 0.001$, ****$p < 0.0001$ by two-way ANOVA with Tukey's multiple comparisons test. In D, $F = 254.1$, $df = 3$. In E, $F = 111.0$, $df = 3$.

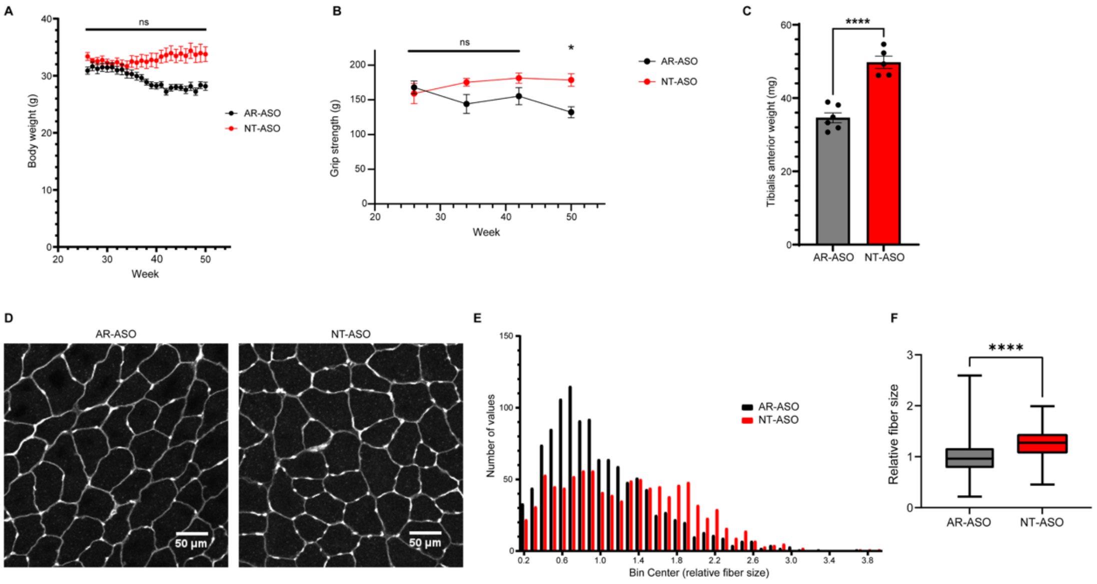

Supplemental Figure 3. Effects of *AR*-targeted and non-targeted ASO in WT male mice.

C57BL/6J males at 26 weeks received subcutaneous administration of *AR*-targeted (n = 6) or non-targeted (NT) ASO (n = 5) (25mg/kg body weight), once per week until 52 weeks.

A. Body weight, assessed weekly. Data are mean ± s.e.m. ns, not significant by two-way ANOVA with Šídák's multiple comparisons test.

B. Grip strength, assessed every two months. Data are mean ± s.e.m. ns, not significant, *$p<0.05$ by two-way ANOVA with Šídák's multiple comparisons test.

C. TA weight at 52 weeks. Data are mean ± s.e.m. ****$p<0.0001$ by unpaired t-test with Welch's correction.

D. TA muscle fibers were visualized by wheat germ agglutinin (WGA)-Alexa Fluor Plus 405. Scale bar = 50 µm.

E., F. Relative TA fiber size quantified as a histogram of frequency distribution (E) and box plot (F) of fiber size, normalized to mean fiber size of AR-ASO treated mice. In panel F, the box is the inter-quartile range, the center line is the median, and the whiskers are the minimum and maximum values. n = 5 mice per group, >100 fibers/mouse, ****p<0.0001 by unpaired t-test with Welch's correction. F = 1.216, df = 438.8.

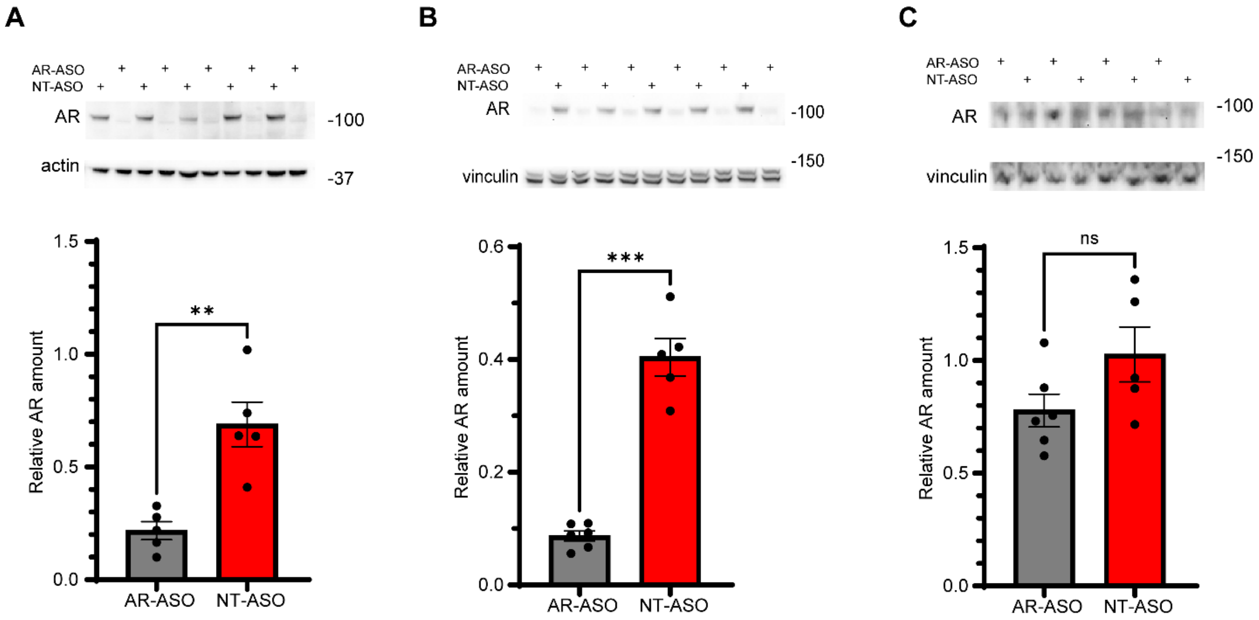

Supplemental Figure 4. *AR*-targeted ASO decreases AR protein levels in peripheral tissues but not in the spinal cord.

C57BL/6J males at 26 weeks received subcutaneous administration of *AR*-targeted or non-targeted (NT) ASO (25mg/kg body weight), once per week until 52 weeks. AR protein levels were determined in liver (A), TA muscle (B), and spinal cord (C) by western blot. Lanes are labeled with a plus sign indicating treatment (*AR*-targeted ASO = AR-ASO; non-targeted ASO = NT-ASO). Protein levels quantified below relative to actin or vinculin. Data are mean ± s.e.m. (A) $^{**}p < 0.01$ by unpaired t test with Welch's correction, $F = 6.036$, $df = 5.290$. (B) $^{***}p < 0.001$ by unpaired t test with Welch's correction, $F = 11.71$, $df = 4.571$. (C) ns = not significant by unpaired t test with Welch's correction, $F = 2.292$, $df = 6.726$.

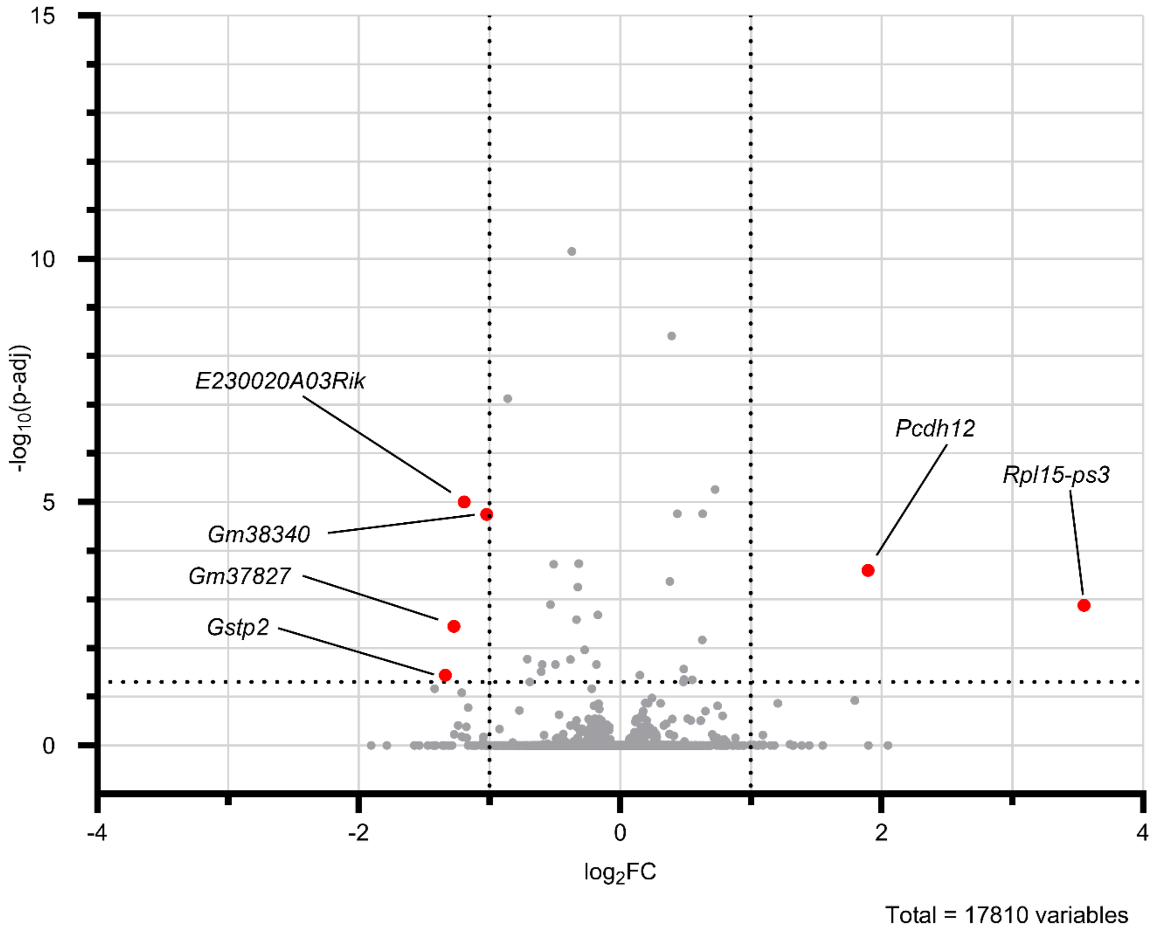

Supplemental Figure 5. Analysis of spinal cord gene expression after peripheral administration of *AR*-targeted ASO.

C57BL/6J males at 26 weeks received subcutaneous administration of *AR*-targeted (n = 6) or non-targeted ASO (n = 5) (25mg/kg body weight), once per week until 52 weeks. Bulk RNA-seq was performed on RNA isolated from lumbar spinal cord. Volcano plot of differentially expressed genes, comparing AR-ASO vs. NT-ASO (control). Dotted lines indicate thresholds for significance (p-adj < 0.05, $|log_2FC| > 1$), with significant differentially expressed genes enlarged and highlighted in red for ease of visibility. Notably, *Ar* itself is not differentially expressed, with $log_2FC = -0.086$ and p-adj = 0.999.

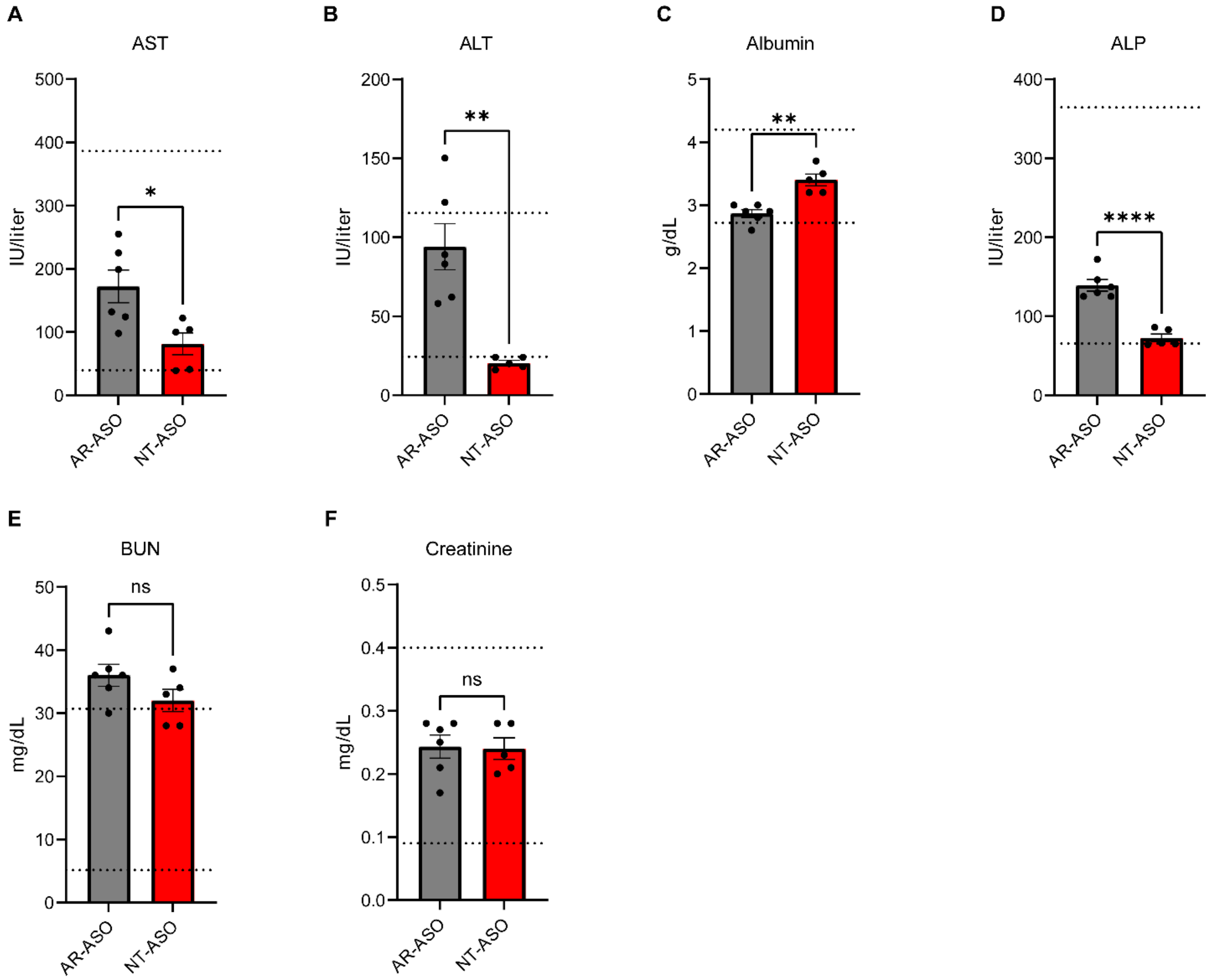

Supplemental Figure 6. Serum markers of liver and kidney function after ASO treatment.

C57BL/6J males at 26 weeks received subcutaneous administration of *AR*-targeted (n = 6) or non-targeted ASO (n = 5) (25mg/kg body weight), once per week until 52 weeks. Serum was collected and analyzed for aspartate aminotransferase (AST) (A), alanine transaminase (ALT) (B), albumin (C), alkaline phosphatase (ALP) (D), blood urea nitrogen (BUN) (E), and creatinine (F). Dotted lines indicate lower and upper bounds of normal range, as provided by the Unit for Laboratory Animal Medicine Pathology Core

at the University of Michigan. Data are mean ± s.e.m. ns, not significant, *$p < 0.05$, **$p < 0.01$, ****$p < 0.0001$ by unpaired t-test with Welch's correction.

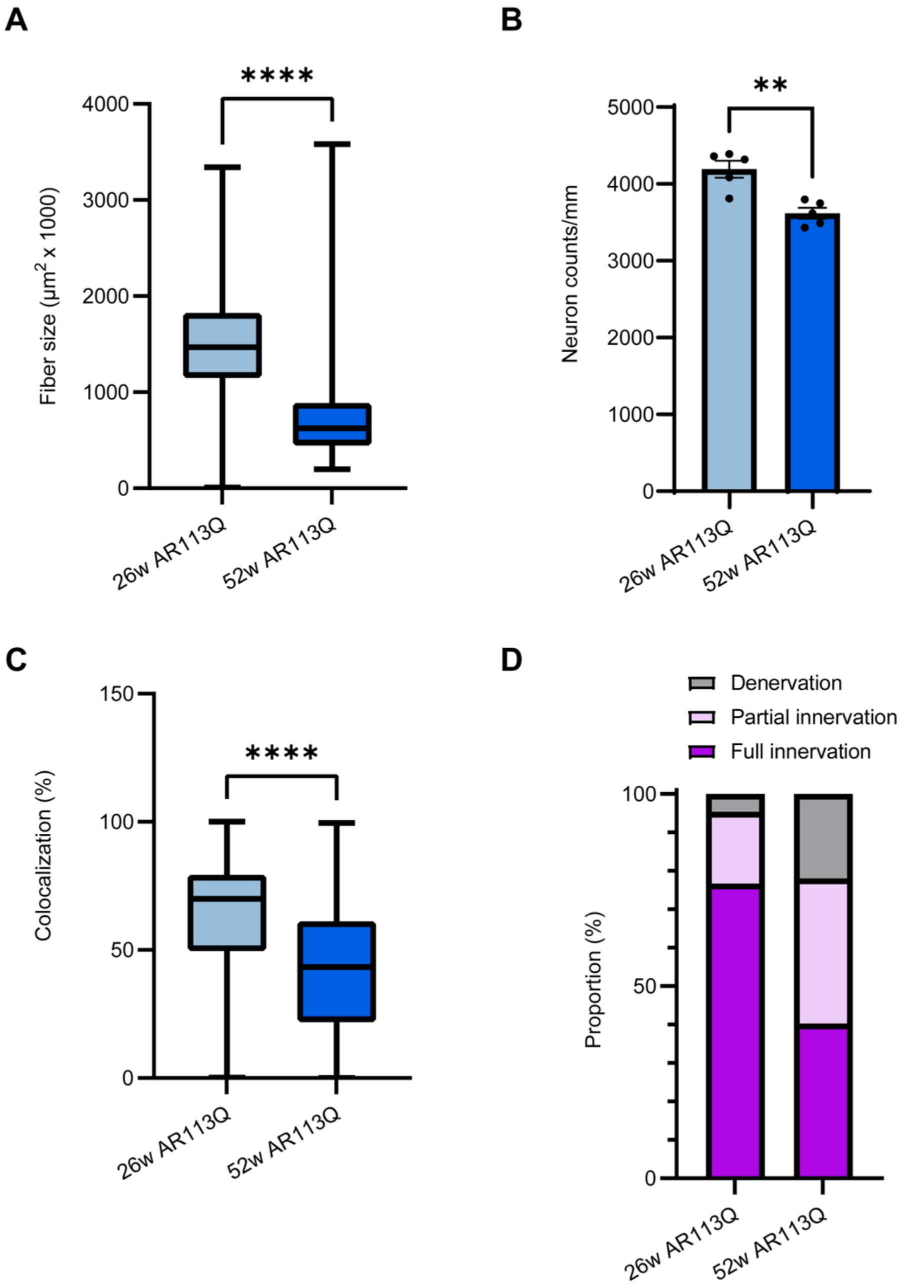

Supplemental Figure 7. Age-dependent progression of neuromuscular pathology in AR113Q male mice.

A. Fiber size in TA muscle from 26- and 52-week male AR113Q mice, shown as a box plot. n = 3 mice/age, >100 fibers/mouse. The box is the inter-quartile range, the center

line is the median, and the whiskers are the minimum and maximum values. ****p < 0.0001 by unpaired *t* test with Welch's correction, F = 2.201, df = 3143.

B. Motor neuron count in anterior spinal cord lumbar enlargement. n = 5 mice/age. Data are mean ± s.e.m. **p < 0.01 by unpaired *t* test with Welch's correction, F = 2.349, df = 6.883.

C., D. NMJ innervation quantified by box plot (C) or stacked bar graph (D). n = 3 mice/age, 100 NMJs/mouse. In panel C, the box is the inter-quartile range, the center line is the median, and the whiskers are the minimum and maximum values. ****p < 0.0001 by unpaired *t* test with Welch's correction, F = 1.095, df = 596.8.

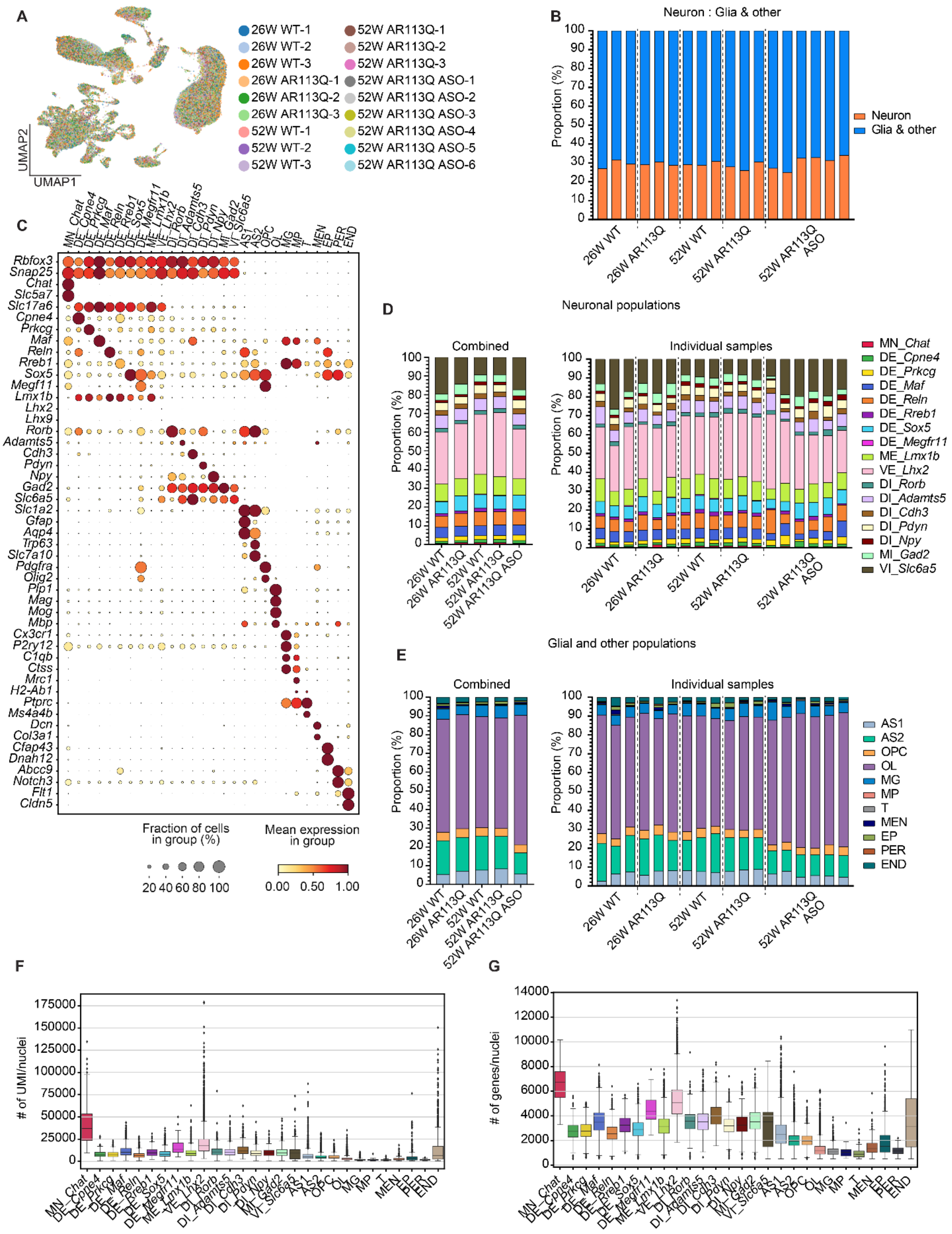

Supplemental Figure 8. Quality control information for snRNA-seq.

A. UMAP embeddings of snRNA-seq data colored by sample to demonstrate integration of data from multiple samples after batch correction (BBKNN).

B. Ratio between neuron and glial plus other cell types by sample.

C. Dot plot displaying mean scaled expression of previously reported marker genes for each cell type used for cell type annotation.

D., E. Relative proportions of each cell type within each animal for neuronal (D) and glial plus other populations (E).

F. Box plots showing average number of unique molecular identifiers (UMIs) per nucleus in each cell type. Boxes indicate the inter-quantile range (IQR), the center line presents the median, whiskers extend to 1.5 times the IQR, and outliers beyond this range are shown as individual points.

G. Box plots of the number of genes per nucleus in cell type. Boxes indicate the IQR, the center line presents the median, whiskers extend to 1.5 times the IQR, and outliers beyond this range are shown as individual points.

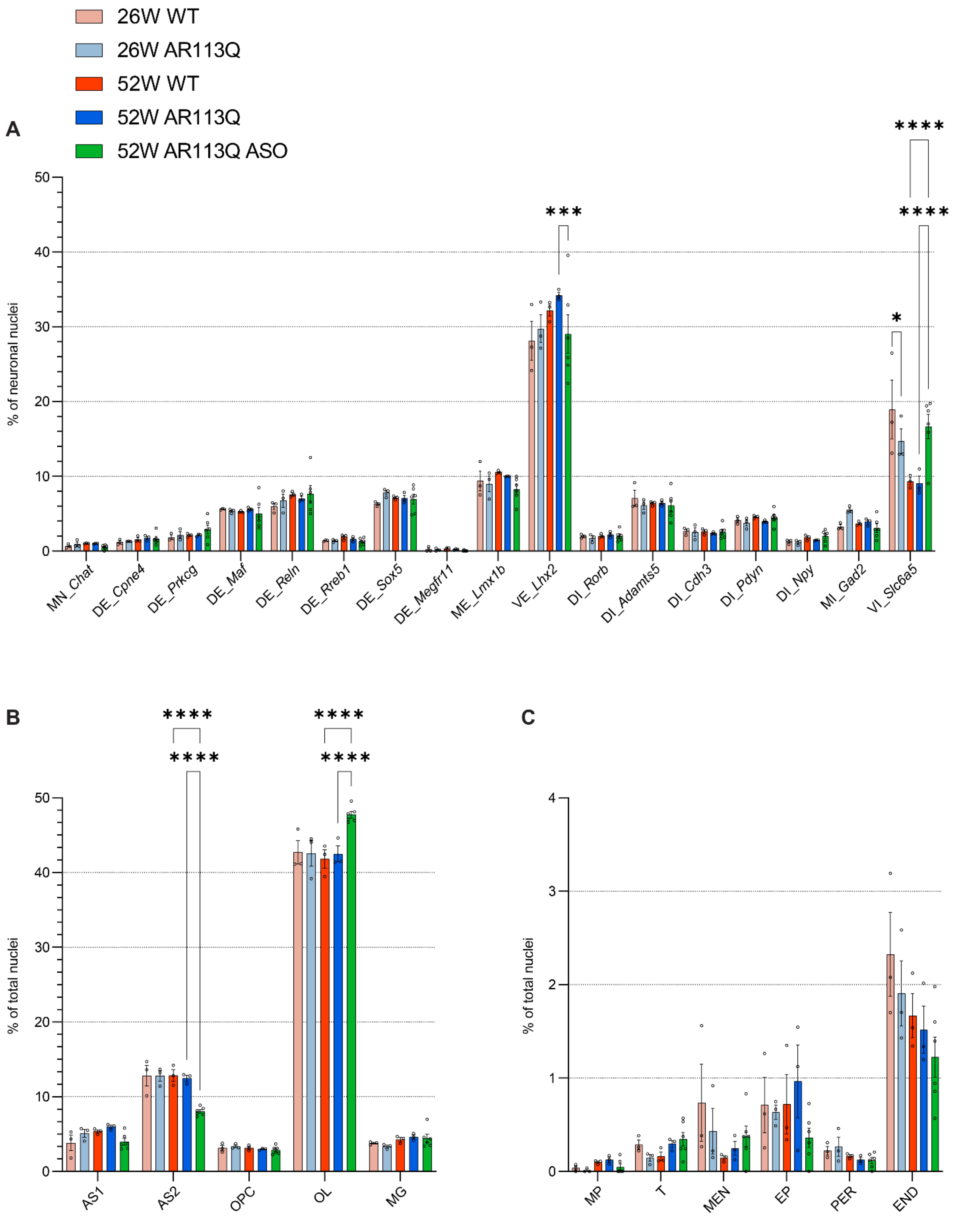

Supplemental Figure 9. Relative abundance of each cell type in each group.

A. Bar plots showing the relative proportions of neuronal cell types within total neuronal nuclei detected.

B., C. Bar plots showing the relative proportions of glial (B) and other (C) cell types within total nuclei detected.

Data shown are mean±s.e.m. Two-way ANOVA were used to compare across groups within a cell type (26W WT, n=3; 26W AR113Q, n=3; 52W WT, n=3, 52W AR113Q, n=3; 52W AR113Q ASO, n=6). Only showing statistically significant comparisons within each timepoint. *$P<0.05$, ***$P<0.005$, ****$P<0.001$.

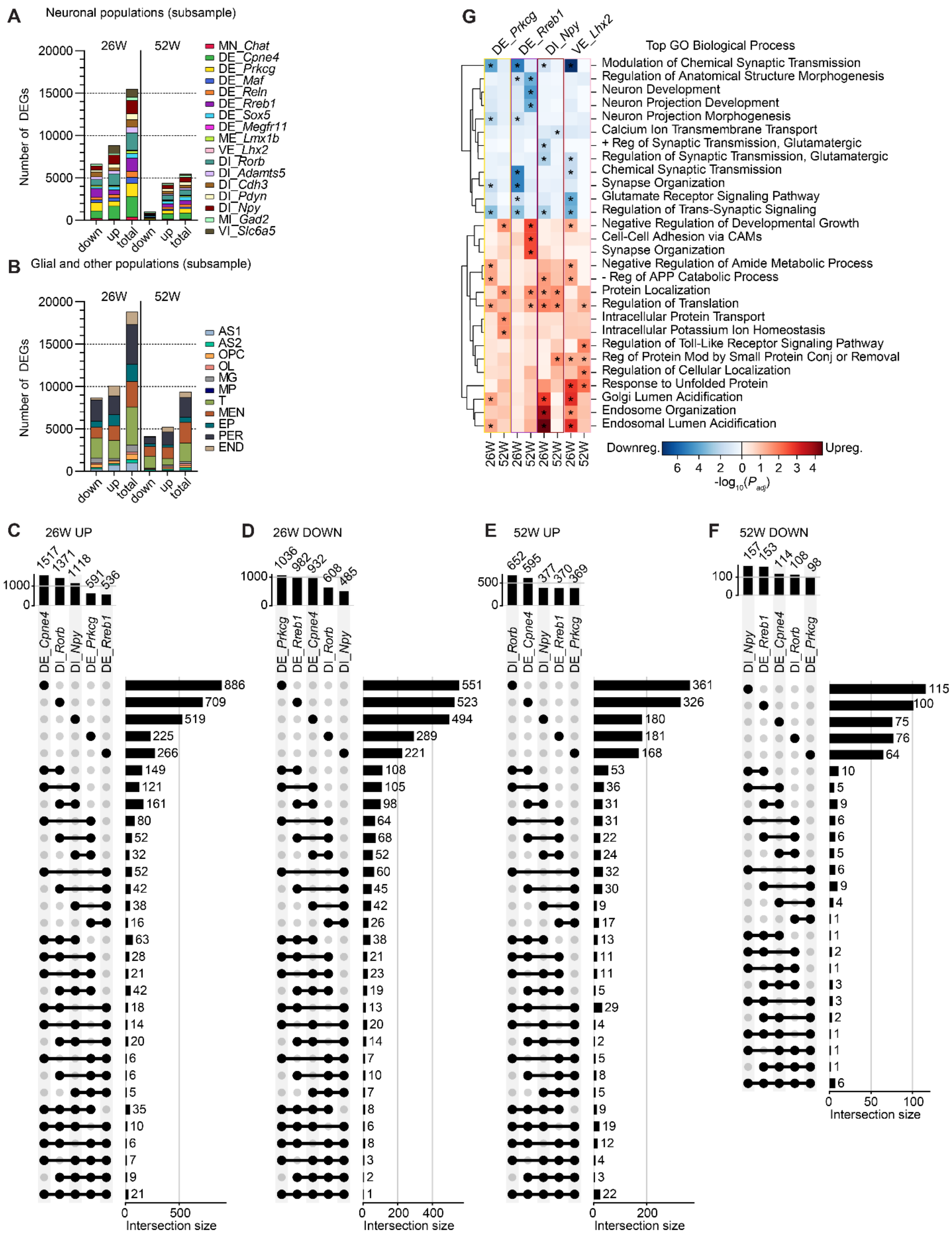
A
Neuronal populations (subsample)
26W
52W
Number of DEGs
MN_Chat
DE_Cpne4
DE_Prkcg
DE_Maf
DE_Reln
DE_Rreb1
DE_Sox5
DE_Megfr11
ME_Lmx1b
VE_Lhx2
DI_Rorb
DI_Adamts5
DI_Cdh3
DI_Pdyn
DI_Npy
MI_Gad2
VI_Slc6a5
down
up
total
B
Glial and other populations (subsample)
AS1
AS2
OPC
OL
MG
MP
T
MEN
EP
PER
END
C
26W UP
D
26W DOWN
E
52W UP
F
52W DOWN
Intersection size
G
DE_Prkcg
DE_Rreb1
DI_Npy
VE_Lhx2
Top GO Biological Process
Modulation of Chemical Synaptic Transmission
Regulation of Anatomical Structure Morphogenesis
Neuron Development
Neuron Projection Development
Neuron Projection Morphogenesis
Calcium Ion Transmembrane Transport
+ Reg of Synaptic Transmission, Glutamatergic
Regulation of Synaptic Transmission, Glutamatergic
Chemical Synaptic Transmission
Synapse Organization
Glutamate Receptor Signaling Pathway
Regulation of Trans-Synaptic Signaling
Negative Regulation of Developmental Growth
Cell-Cell Adhesion via CAMs
Synapse Organization
Negative Regulation of Amide Metabolic Process
- Reg of APP Catabolic Process
Protein Localization
Regulation of Translation
Intracellular Protein Transport
Intracellular Potassium Ion Homeostasis
Regulation of Toll-Like Receptor Signaling Pathway
Reg of Protein Mod by Small Protein Conj or Removal
Regulation of Cellular Localization
Response to Unfolded Protein
Golgi Lumen Acidification
Endosome Organization
Endosomal Lumen Acidification
Downreg.
Upreg.
-log10(Padj)

Supplemental Figure 10. Analyses of DEGs in the subsampled dataset and comparison across timepoints.

A., B. Number of downregulated, upregulated, and total DEGs (imputed |EMD|≥0.1 and $P_{corrected}$<0.01) in neurons (A) and glial and other populations (B) after subsampling such that equal number of nuclei are compared across all groups.

C.-F. UpSet plots illustrating the numbers of DEGs in DE_*Cpne4*, DE_*Prkcg*, DE_*Rreb1*, DI_*Rorb*, and DI_*Npy*, along with their overlap among these gene sets of upregulated (C, E) and downregulated (D, F) DEGs at 26 and 52 weeks. Vertical bar graphs display total genes in each set, and horizontal bar graphs display unique and overlapping genes with separate and linked dots on each row.

G. Heatmap of GO biological process analysis for downregulated (top) and upregulated (bottom) DEGs in DE_*Prkcg*, DE_*Rreb1*, DI_*Npy*, and VE_*Lhx2* at 26 and 52 weeks. The top three significantly enriched GO terms for each gene set are shown. Asterisks indicate statistically significant enrichment (adjusted P<0.05).

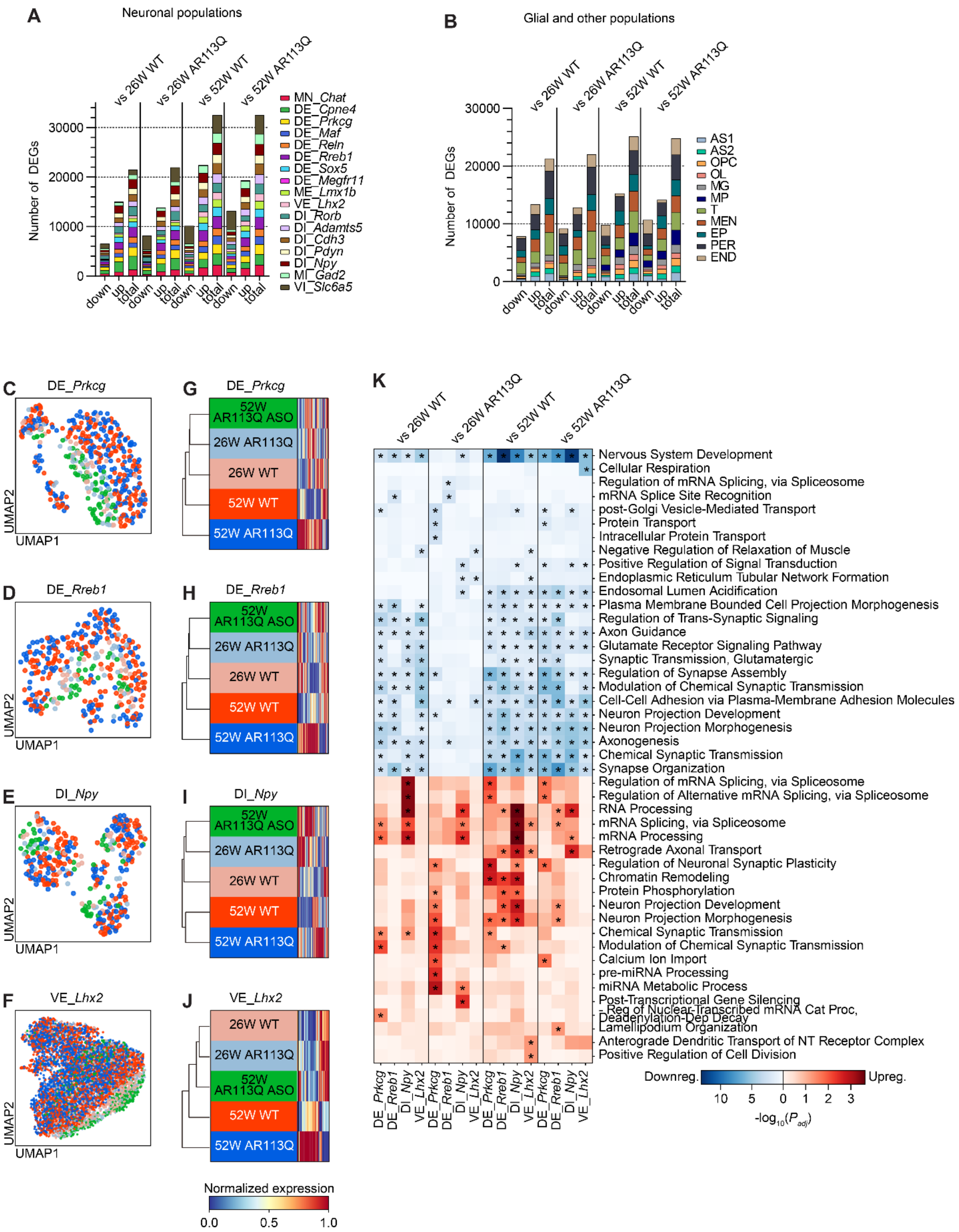

A
Neuronal populations
vs 26W WT
vs 26W AR113Q
vs 52W WT
vs 52W AR113Q
Number of DEGs
0
10000
20000
30000
down
up
total
MN_Chat
DE_Cpne4
DE_Prkcg
DE_Maf
DE_Reln
DE_Rreb1
DE_Sox5
DE_Megfr11
ME_Lmx1b
VE_Lhx2
DI_Rorb
DI_Adamts5
DI_Cdh3
DI_Pdyn
DI_Npy
MI_Gad2
VI_Slc6a5
B
Glial and other populations
vs 26W WT
vs 26W AR113Q
vs 52W WT
vs 52W AR113Q
Number of DEGs
0
10000
20000
30000
down
up
total
AS1
AS2
OPC
OL
MG
MP
T
MEN
EP
PER
END
C
DE_Prkcg
UMAP2
UMAP1
D
DE_Rreb1
UMAP2
UMAP1
E
DI_Npy
UMAP2
UMAP1
F
VE_Lhx2
UMAP2
UMAP1
G
DE_Prkcg
52W AR113Q ASO
26W AR113Q
26W WT
52W WT
52W AR113Q
H
DE_Rreb1
52W AR113Q ASO
26W AR113Q
26W WT
52W WT
52W AR113Q
I
DI_Npy
52W AR113Q ASO
26W AR113Q
26W WT
52W WT
52W AR113Q
J
VE_Lhx2
26W WT
26W AR113Q
52W AR113Q ASO
52W WT
52W AR113Q
Normalized expression
0.0
0.5
1.0
K
vs 26W WT
vs 26W AR113Q
vs 52W WT
vs 52W AR113Q
Nervous System Development
Cellular Respiration
Regulation of mRNA Splicing, via Spliceosome
mRNA Splice Site Recognition
post-Golgi Vesicle-Mediated Transport
Protein Transport
Intracellular Protein Transport
Negative Regulation of Relaxation of Muscle
Positive Regulation of Signal Transduction
Endoplasmic Reticulum Tubular Network Formation
Endosomal Lumen Acidification
Plasma Membrane Bounded Cell Projection Morphogenesis
Regulation of Trans-Synaptic Signaling
Axon Guidance
Glutamate Receptor Signaling Pathway
Synaptic Transmission, Glutamatergic
Regulation of Synapse Assembly
Modulation of Chemical Synaptic Transmission
Cell-Cell Adhesion via Plasma-Membrane Adhesion Molecules
Neuron Projection Development
Neuron Projection Morphogenesis
Axonogenesis
Chemical Synaptic Transmission
Synapse Organization
Regulation of mRNA Splicing, via Spliceosome
Regulation of Alternative mRNA Splicing, via Spliceosome
RNA Processing
mRNA Splicing, via Spliceosome
mRNA Processing
Retrograde Axonal Transport
Regulation of Neuronal Synaptic Plasticity
Chromatin Remodeling
Protein Phosphorylation
Neuron Projection Development
Neuron Projection Morphogenesis
Chemical Synaptic Transmission
Modulation of Chemical Synaptic Transmission
Calcium Ion Import
pre-miRNA Processing
miRNA Metabolic Process
Post-Transcriptional Gene Silencing
- Reg of Nuclear-Transcribed mRNA Cat Proc, Deadenylation-Dep Decay
Lamellipodium Organization
Anterograde Dendritic Transport of NT Receptor Complex
Positive Regulation of Cell Division
DE_Prkcg
DE_Rreb1
DI_Npy
VE_Lhx2
Downreg.
Upreg.
10
5
0
1
2
3
-log10(Padj)

Supplemental Figure 11. Effect of peripheral ASO injection on spinal cord cell types.

A., B. Number of downregulated, upregulated, and total DEGs (imputed |EMD|≥0.1 and $P_{corrected}$<0.01) in neurons (A) and glial and other populations (B) between the 52-week AR113Q ASO group and other groups.

C.-F. UMAP embeddings showing DE_*Prkcg* (C), DE_*Rreb1* (D), DI_*Npy* (E), and VE_*Lhx2* (F).

G.-J. Heatmaps with dendrogram showing normalized expression of the respective total DEGs (columns) in DE_*Prkcg* (G), DE_*Rreb1* (H), DI_*Npy* (I), and VE_*Lhx2* (J) between 52-week AR113Q and 52-week WT.

K. Heatmap of GO biological process analysis for downregulated (top) and upregulated (bottom) DEGs in DE_*Prkcg*, DE_*Rreb1*, DI_*Npy*, and VE_*Lhx2* between the 52-week AR113Q ASO group and other groups. The top three significantly enriched GO terms for each gene set are shown. Asterisks indicate statistically significant enrichment (adjusted *P*<0.05).

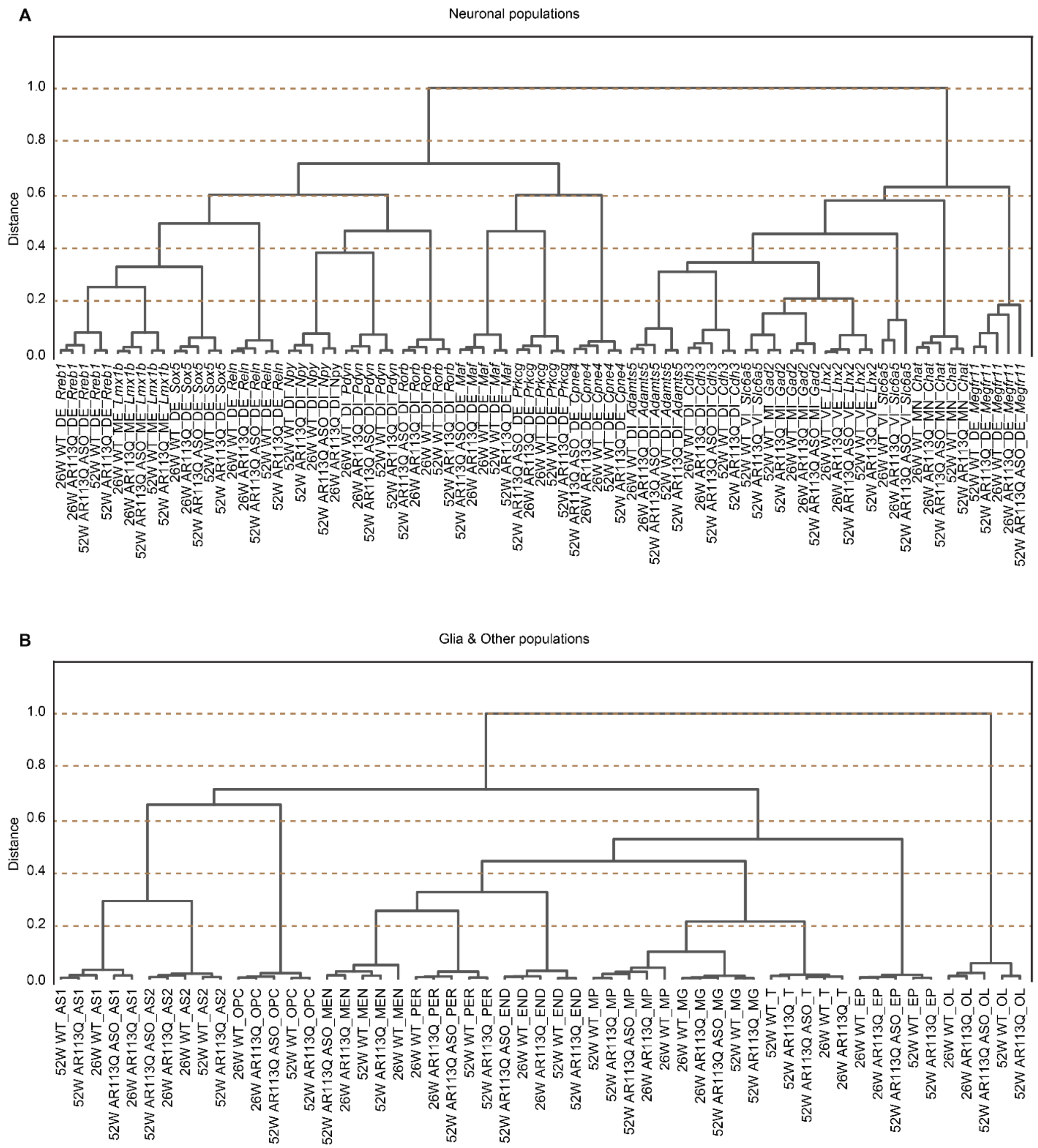

Supplemental Figure 12. Hierarchical clustering of all cell types across all groups based on the entire transcriptional profiles.

A., B. Hierarchical clustering plots showing group-specific cell types on the x-axis and normalized correlation distance based on the transcriptional profiles on the y-axis, for neuronal populations (A) and glial and other populations (B).
