# Supplementary material for: Peripherally administered androgen receptor–targeted antisense oligonucleotide rescues spinal pathology in a murine SBMA model
Source: J Clin Invest. 2025 Aug 28;135(21):e182955. doi: 10.1172/JCI182955 (PMC12578385; doi:10.1172/JCI182955)

S4a: Liver

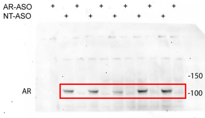

S4a: Liver

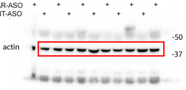

S4b: Tibialis anterior

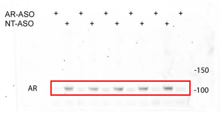

S4b: Tibialis anterior

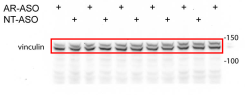

S4c: Spinal cord

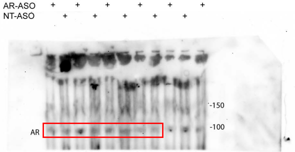

S4c: Spinal cord

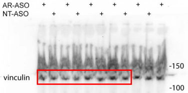

Supplement: Unedited blot and gel images [file jci-135-182955-s009.pdf]
